# Motor Cortex Hyperexcitability Is Coupled to Neuromuscular Dysfunction in Aged Mice

**DOI:** 10.1111/acel.70731

**Published:** 2026-09-23

**Authors:** Jose A. Viteri, Nathan R. Kerr, Fereshteh B. Darvishi, Anna Roshani Dashtmian, Charles D. Brennan, Sindhuja N. Ayyagari, Peter J. Moore, Meifang Wang, Harper Snyder, Baocong Yu, Joseph M. Santin, W. David Arnold

**Affiliations:** ^1^ Department of Physical Medicine and Rehabilitation University of Missouri‐Columbia Columbia Missouri USA; ^2^ NextGen Precision Health University of Missouri‐Columbia Columbia Missouri USA; ^3^ Division of Biological Sciences University of Missouri‐Columbia Columbia Missouri USA

**Keywords:** aging, hyperexcitability, motor cortex, neuromuscular, sarcopenia

## Abstract

Age‐related weakness is strongly associated with disability and mortality in older adults. While muscle atrophy contributes to weakness, strength declines at a faster rate than muscle mass, implicating neural mechanisms such as changes at the spinal cord and neuromuscular junction. Yet, the role of the motor cortex in age‐related weakness remains largely unexplored. We previously identified layer V pyramidal neurons (LVPNs) of the primary motor cortex as hyperexcitable in aged mice, but whether this phenotype was coupled to neuromuscular dysfunction was unknown. Here, we show that aged mice exhibit impaired strength, coordination, and neuromuscular excitability. However, cortical motor output to muscle measured in vivo was enhanced, with patch‐clamp recordings from the same aged animals confirming LVPN hyperexcitability. This was accompanied by altered excitatory and inhibitory synaptic responses evoked by layer II/III stimulation, and by transcriptional changes in excitability‐related genes of aged LVPNs. Statistical analysis across 250 cortical–neuromuscular/behavioral pairwise relationships revealed that greater cortical excitability is broadly and consistently correlated with worse whole‐animal neuromuscular and behavioral outcomes. Most strikingly, LVPN firing frequency emerged as the single strongest correlate of neuromuscular dysfunction and statistically accounted for 75% of the age effect on neuromuscular function, while the reverse analysis showed that neuromuscular dysfunction accounted for 42.2% of the age effect on LVPN firing frequency. These findings identify the motor cortex as a potential contributor to age‐related weakness—a finding with broader significance given that hyperexcitability of LVPNs is a shared feature of motor dysfunction across neurodegenerative disease contexts.

## Graphical abstract

Aged mice exhibited impaired motor behavior and neuromuscular hypoexcitability, yet cortical output to muscle was enhanced, and motor cortex layer V pyramidal neurons were hyperexcitable, with altered synaptic inputs and excitability‐related transcriptional changes. Across 250 cortical–neuromuscular/behavioral pairwise relationships, greater LVPN excitability was associated with worse neuromuscular and behavioral outcomes. LVPN firing frequency emerged as the strongest correlate of neuromuscular dysfunction, statistically accounting for 75% of the age effect on neuromuscular dysfunction—identifying the motor cortex as a candidate contributor to age‐related weakness.

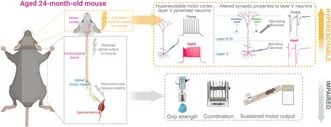

## 1 Introduction

As humans age, strength progressively declines (Dodds et al. 2014; Goodpaster et al. 2006). Importantly, large population studies demonstrate that age‐related weakness is strongly associated with mortality, including deaths from cardiovascular events, cancer, and dementia (Boyle et al. 2009; Yuan and Larsson 2023), highlighting weakness as a major clinical feature of aging. As the global population aged 65 and older is projected to exceed 20% of the world's population by 2050 (Dogra et al. 2022), the burden of age‐related weakness on healthcare systems and individual independence will only increase.

The mechanisms driving this decline are not fully understood. While muscle atrophy has long been considered the primary contributor, strength declines at a substantially faster rate than muscle mass (Goodpaster et al. 2006), indicating that additional mechanisms are at play. The nervous system—which is required to generate and sustain muscular force—is increasingly recognized as a critical contributor to age‐related weakness (Clark 2023). Most work in this area has focused on the spinal cord and periphery, where changes in spinal circuit excitability (Kerr et al. 2025), motor neuron intrinsic properties (Orssatto et al. 2023), synaptic balance (Castro et al. 2023), neuromuscular junction (NMJ) transmission (Arnold and Clark 2023), and NMJ remodeling (Deschenes et al. 2022) are well characterized.

However, how the motor cortex—the cortical source of voluntary motor output—mechanistically contributes to weakness in aging remains largely unexplored (Clark 2023). The existing evidence comes primarily from human studies, pointing to decreased voluntary activation of limb muscles (Clark et al. 2015), altered motor cortex output to muscle, and excitatory/inhibitory (E/I) circuit imbalances in the motor cortex of older adults (OAs) (Clark 2023). Despite the important contribution of these studies, they lack sufficient resolution to identify specific cortical motor circuits, neuronal populations, or molecular mechanisms that change with aging, or to link these changes back to motor dysfunction. Given the absence of approved therapies for age‐related weakness, addressing this mechanistic gap at the level of the motor cortex is critical.

Recently, we identified layer V pyramidal neurons (LVPNs) of the primary motor cortex—a heterogeneous population of projection neurons that includes cells involved in transmitting and shaping cortical motor commands (Grimsley et al. 2013)–as hyperexcitable in aged mice (Viteri, Bueschke, et al. 2025), representing the first identified neuronal population in the motor cortex with altered excitability in aging. This phenotype is striking because it mirrors that seen in neurodegenerative diseases such as amyotrophic lateral sclerosis (ALS) and Alzheimer's disease (AD) (Menon et al. 2015; Targa Dias Anastacio et al. 2022), where cortical hyperexcitability has been implicated in motor and cognitive decline, respectively. Thus, we tested the hypothesis that motor cortex hyperexcitability is coupled to motor dysfunction in aging. Accordingly, we fully characterized the neuromuscular phenotype of aged mice—including motor behavior, in vivo nerve and muscle electrophysiology, and muscle contractility—alongside layer V patch‐clamp recordings of intrinsic excitability and synaptic inputs to examine how cortical properties relate to neuromuscular outcomes across the same animals.

Here, we show that neuromuscular dysfunction is strongly coupled to enhanced cortical motor output and to hyperexcitability of motor cortex layer V neurons. LVPN firing frequency emerged as the strongest cortical correlate of neuromuscular dysfunction and statistically accounted for 75% of the age effect on neuromuscular function, while the reverse analysis showed that neuromuscular dysfunction accounted for 42.2% of the age effect on LVPN firing frequency. This hyperexcitability was accompanied by changes in excitatory and inhibitory synaptic responses evoked by layer II/III stimulation and transcriptional changes in excitability‐related genes within LVPNs, identifying the motor cortex as a potential contributor to age‐related weakness.

## 2 Methods

### 2.1 Animals

All animal experiments were approved by the Institutional Animal Care and Use Committee (IACUC) at the University of Missouri—Columbia (protocol #39905). C57BL/6N mice (Charles River Laboratories) were used for all experiments, with cohorts of either 3 months ±20 days (young; *N* = 16, 50% female) or 24 months ±25 days (aged; *N* = 16, 50% female) of age. For all in vivo neuromuscular excitability and motor behavior assessments, “*N*” refers to mouse sample size. The same young (*N* = 16, *n* = 77) and aged (*N* = 16, *n* = 83) mice used for in vivo electrophysiology and motor behavior assessments subsequently underwent in vitro whole‐cell patch clamp recordings from layer V of the primary motor cortex, where “*n*” refers to the number of individual recordings (see *Patch Clamp Electrophysiology* below).

### 2.2 Motor Behavior Assessments

**FIGURE 1:**
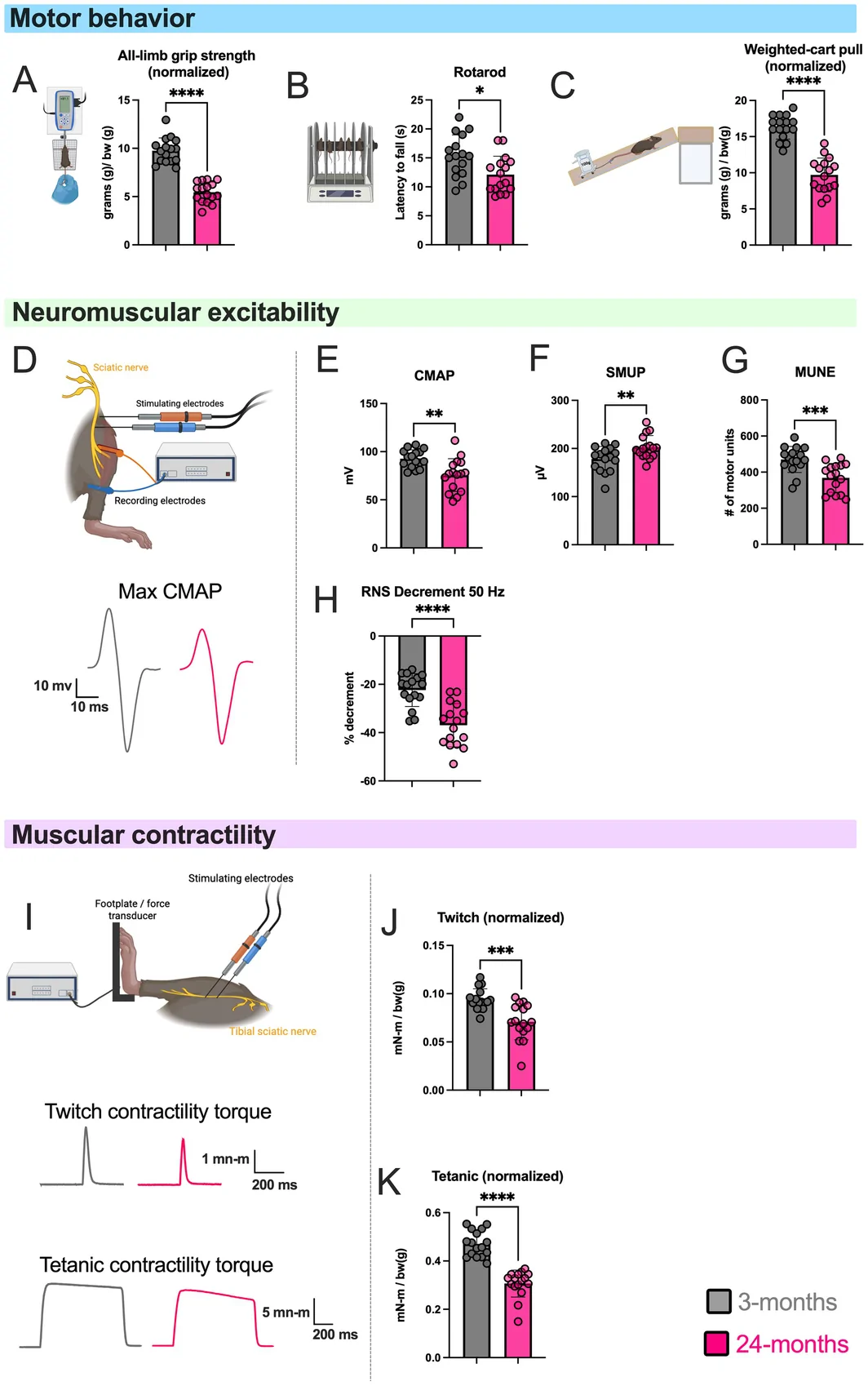
Aged mice are weak and exhibit impairments in neuromuscular excitability and contractile function. (A–C) Grip strength, rotarod latency to fall, and weighted cart pull were all reduced in aged mice compared to young. (D–H) Aged mice exhibited reduced CMAP amplitude and MUNE, increased SMUP amplitude, and a greater RNS % decrement at 50 Hz compared to young mice. Representative CMAP traces are shown in (D). (I–K) Both twitch and tetanic plantar flexion torque were reduced in aged mice compared to young. Representative twitch and tetanic contractile traces are shown in (I). Significance: *p < 0.05, **p < 0.01, ***p < 0.001, ****p < 0.0001. Data are presented as means ± SD. *N* = 16 mice per group (50% female). *Figure made using BioRender*.

All motor behavior assessments were performed as previously described (Kerr et al. 2025; Viteri, Kerr, et al. 2025). Some of these assessments were normalized to body weight. The raw non‐normalized data for grip strength and the weighted‐cart pull assessments are included in Figure S1. Schematics of the setups are shown in Figure 1A–C.

#### 2.2.1 All‐Limb Grip Strength

All‐limb grip strength was measured using a grip force transducer (Bioseb GT3, Pinellas Park, FL, USA). The experimenter held the mouse by the tail, placed it on a metal grid attached to the force transducer, and ensured all four limbs were grasping the grid. A steady horizontal pull was then applied until all limbs lost contact with the grid. Three trials were recorded per mouse, and the final value was the average of the three trials normalized to body weight (grams/g body weight). Values were normalized to body weight because body weight can differ substantially between young and aged mice, as well as between males and females, and grip strength is a direct force output measure expected to scale with body mass.

#### 2.2.2 Rotarod

Motor coordination was assessed using an accelerating rotarod (BX‐ROD, Bioseb, Pinellas Park, FL, USA). Mice were placed on the rotating rod and allowed to freely walk for 30 s at 4 rpm. The rod then accelerated from 4 to 40 rpm over 60 s. Latency to fall was recorded for each trial. Three trials were performed, and the final value was the average latency. Rotarod performance was not normalized to body weight, as this test depends on coordination, motivation, and balance in addition to strength, and is not a direct force output measure expected to scale with body mass.

#### 2.2.3 Weighted Cart Pull

Maximum whole‐body functional strength was assessed using an adapted version of the weighted cart pull assay we previously developed in Brennan et al. (Brennan et al. 2025). A weighted cart was attached to the mouse's tail and the mouse was placed at the bottom of an inclined track set at a 10° incline, with a dark goal box at the top. Each session began with three warm‐up trials at 5 g/g body weight. The first experimental trial began at 10 g/g body weight; if the mouse completed the full track, the load was increased by 1 g/g body weight per trial, with a 2‐min rest between trials. For mice unable to complete 10 g/g, weight was progressively reduced to find their maximum. Failure was defined as inability to complete the full track or sliding back down. If the mouse paused for more than 5 s, gentle prodding was applied to the rear. All mice were acclimated to the task prior to testing. The maximum weight successfully pulled, normalized to body weight, was used as the final value. Values were normalized to body weight for the same reason as grip strength: this is a force output measure expected to scale with body mass, and normalization controls for the weight difference between young and aged mice.

### 2.3 In Vivo Neuromuscular Electrophysiology

All in vivo neuromuscular electrophysiology experiments were performed as previously described (Kerr et al. 2025; Viteri, Kerr, et al. 2025). Compound muscle action potential (CMAP; the summed electrical response recorded from a muscle and used as a measure of overall neuromuscular excitability), single motor unit potential (SMUP; used to estimate the average size of single motor units), repetitive nerve stimulation % decrement (RNS; used to detect neuromuscular junction transmission deficits), and motor unit number estimation (MUNE) were recorded from the gastrocnemius. A schematic of the setup is shown in Figure 1D.

Mice were anesthetized with ketamine (100 mg/kg) and xylazine (7.5 mg/kg) by intraperitoneal injection. Animals were placed prone on a 37°C heating pad and the right hindlimb was taped so that it formed ~90° relative to the tail. Eye lubricant was applied to prevent drying of the eyes. Two gel‐coated ring electrodes (MFI Medical, San Diego, CA, USA) were placed around the gastrocnemius (Electrode 1) and the calcaneus (Electrode 2). Stimulating needle electrodes (anode and cathode) were inserted subcutaneously near the sciatic nerve, and a ground electrode was placed on the tail. Recordings were acquired using the Sierra Summit system (Cadwell, Kennewick, WA, USA).

For CMAP recordings, a supramaximal stimulus was delivered to the sciatic nerve, and responses were measured as peak‐to‐peak amplitudes. Screen sensitivity was set to 10 mV/division and sweep duration was 10 ms (1 ms/division).

SMUP amplitude was determined using incremental stimulation delivered at 1 Hz. Stimulus intensity was gradually increased until 10 all‐or‐none increments were obtained. The amplitude of the tenth increment was divided by 10 to estimate the average SMUP amplitude. Screen parameters for SMUP recordings were 200 μV/division with a sweep duration of 1 ms/division.

MUNE was calculated using the standard equation: MUNE = CMAP amplitude/SMUP amplitude.

Neuromuscular junction transmission was assessed using repetitive nerve stimulation. Ten supramaximal stimuli were delivered at 50 Hz and CMAP responses were recorded. Decrement was calculated as:

$$\left[\left(\text{Amplitude of}\;10\mathrm{th}\;\text{CMAP}-\text{Amplitude of 1st CMAP}\right)/\text{Amplitude of 1st CMAP}\right]\times 100\%.$$

**FIGURE 2:**
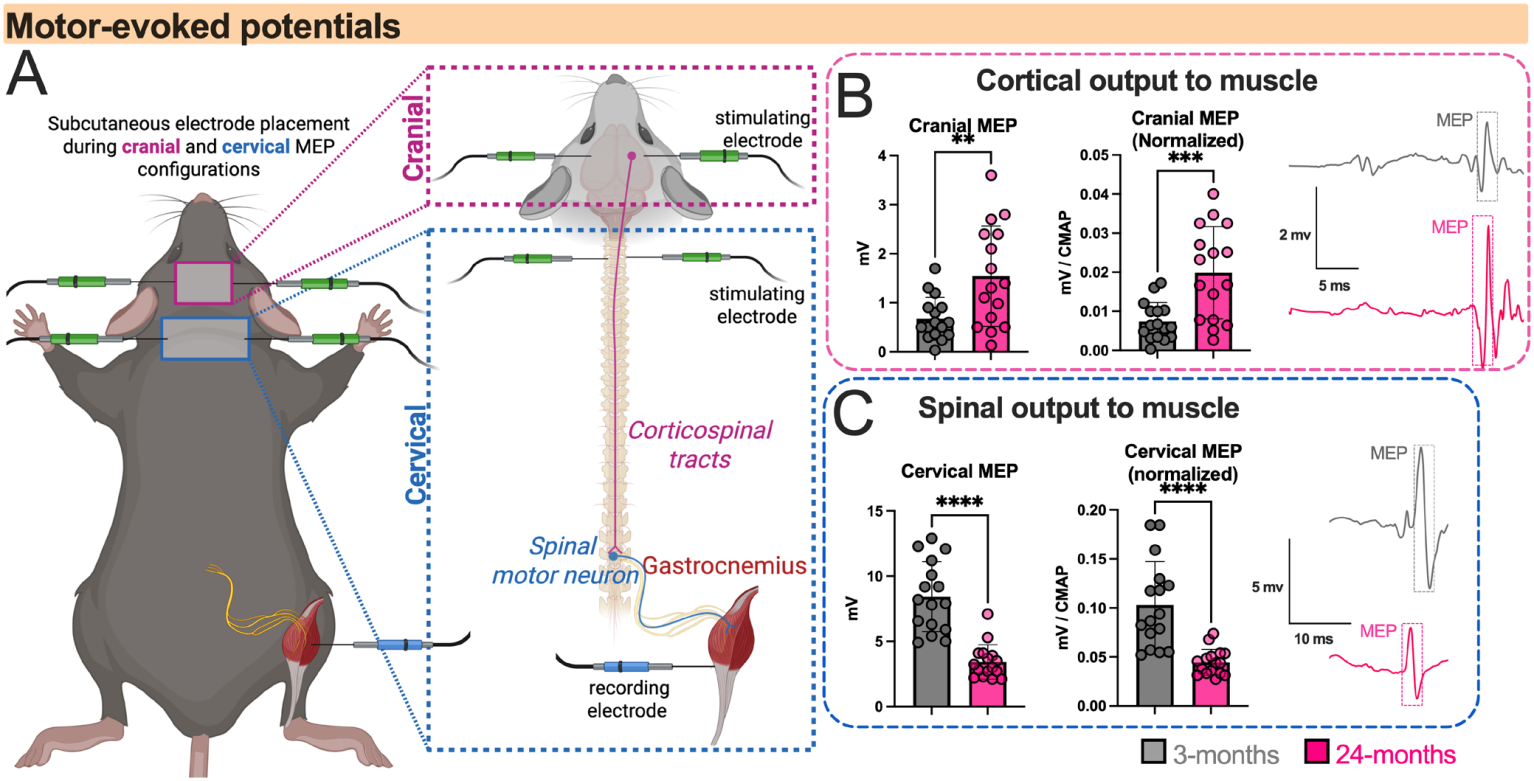
Aged mice exhibit enhanced cortical output to muscle. (A) Schematic illustrating the two stimulation configurations, showing the cranial and cervical pathways being stimulated and gastrocnemius recording. (B) Raw and CMAP‐normalized cranial MEP amplitude was increased in aged mice; representative traces are shown. (C) Raw and CMAP‐normalized cervical MEP amplitude was reduced in aged mice; representative traces are shown. Significance: **p < 0.01, ***p < 0.001, ****p < 0.0001. Data are presented as means ± SD. *N* = 16 mice per group. *Figure made using BioRender*.

Motor evoked potentials (MEPs) are compound muscle responses produced by cranial or cervical stimulation and provide a measure of cortical or spinal output (Kerr et al. 2025; Viteri, Kerr, et al. 2025; Ketabforoush et al. 2026). The recording configuration was the same as for CMAP recordings, with differences only in the location of the stimulating electrodes (see Figure 2A for schematic and representative electrode placement). For cranial MEPs, stimulating electrodes (MFI Medical, San Diego, CA, USA) were inserted subcutaneously approximately 3–4 mm lateral to the bregma on opposite sides of the skull (Figure 2A). With this placement, current passes bilaterally across the motor cortex, generating a broad electrical field capable of recruiting motor cortical output that, per our prior work, can be recorded bilaterally from both limbs (Ketabforoush et al. 2026); here MEPs were recorded unilaterally (Kerr et al. 2025; Viteri, Kerr, et al. 2025). For cervical MEPs, stimulating electrodes were placed subcutaneously near the C2–C3 vertebrae, caudal to the ears, on opposite sides of the spinal column. With this placement, current passes bilaterally across the cervical spinal cord, generating a broad electrical field capable of stimulating corticospinal tracts; the resulting MEPs were recorded unilaterally from the gastrocnemius (Kerr et al. 2025; Viteri, Kerr, et al. 2025). For both cranial and cervical MEPs, the stimulus duration was 0.2 ms, and the stimulus intensity was increased from 15 to 60 mA in 5 mA increments, with electrodes repositioned as needed, until the MEP amplitude no longer increased with further increases in intensity, indicating a maximal response. Responses were recorded from the gastrocnemius. Latencies were used to verify the response, with cranial MEPs typically 15–17 ms and cervical MEPs 5–10 ms, consistent with our prior work (Kerr et al. 2025; Viteri, Kerr, et al. 2025; Ketabforoush et al. 2026). MEP sensitivity was set to 500 μV/division and sweep duration to 5 ms/division. Final MEP values were normalized to CMAP amplitude (MEP amplitude/CMAP amplitude) to account for differences in neuromuscular excitability. Non‐normalized raw MEP values are also included in Figure 2.

### 2.4 Muscle Contractility

Muscle contractility experiments were performed as described (Kerr et al. 2025; Viteri, Kerr, et al. 2025). Plantar flexion torque was measured to assess neuromuscular output and muscle contractile capacity following peripheral nerve stimulation. A schematic of the setup is shown in Figure 1I. After completing CMAP and MEP recordings, the right foot was secured to a force pedal using medical tape. The tibia was positioned at ~90° relative to the femur. The femoral condyles were clamped so that movement occurred only at the ankle joint. Stimulating electrodes (MFI Medical, San Diego, CA, USA) were inserted subcutaneously near the tibial branch of the sciatic nerve. Force recordings were obtained using the 1305A whole animal system (Aurora Scientific Inc., Canada). Twitch torque was produced using a single supramaximal pulse (0.20 ms). Tetanic torque was produced using a 1‐s stimulus train at 125 Hz. Torque output was recorded in millinewton‐meters (mN‐m) and normalized to body weight.

### 2.5 Patch‐Clamp Electrophysiology

Whole‐cell patch clamp recordings were performed in LVPNs of the primary motor cortex. The motor cortex and LVPNs were identified as described in Viteri, Bueschke, et al. (2025) Viteri, Kerr, et al. (2025). A limitation of this approach is that recorded LVPNs were not retrogradely or genetically identified by projection class. Therefore, the population sampled here may include both pyramidal‐tract/corticospinal (PT/CSN) and intratelencephalic (IT) neurons.

All patch‐clamp experimental methods below were performed similarly to protocols previously described in Viteri, Bueschke, et al. (2025), Viteri, Kerr, et al. (2025). All current‐clamp and voltage‐clamp recordings were performed sequentially in the same neuron when possible. The order of protocols was F‐I recordings, followed by excitatory postsynaptic currents (EPSC) paired‐pulse ratio (PPR), 0.5 Hz EPSC recordings, inhibitory postsynaptic currents (IPSC) PPR, and 0.5 Hz IPSC recordings. Offline analysis of recordings was performed using Easy Electrophysiology software (www.easyelectrophysiology.com).

#### 2.5.1 Brain Slice Preparation

Acute brain slices were prepared using a modified N‐methyl‐D‐glucamine (NMDG)‐recovery approach as previously described (Viteri, Bueschke, et al. 2025; Viteri, Kerr, et al. 2025). Mice were deeply anesthetized with isoflurane and decapitated once the toe‐pinch reflex was absent. Brains were quickly removed and placed in ice‐cold oxygenated NMDG‐ACSF (in mM: 92 NMDG, 2.5 KCl, 20 HEPES, 30 NaHCO₃, 1.25 NaH_2_PO₄, 25 D‐glucose, 0.5 CaCl_2_·2H_2_O [500 μL from 1 M stock], 10 MgCl_2_·2H_2_O [10 mL from 1 M stock], 2 thiourea, 5 Na‐ascorbate, and 3 Na‐pyruvate). Solutions were equilibrated to pH 7.3–7.4 using 95% O_2_/5% CO_2_ (Millipore Sigma, Burlington, MA, USA; Fisher Scientific, Hampton, NH, USA).

Coronal sections (300 μm) containing the primary motor cortex were cut using a vibratome (EMS 7000; frequency 70 Hz, amplitude 1.50 mm, advance speed 0.10 mm/s). Slices were transferred to warmed NMDG‐ACSF (34°C–35°C) for 30 min. After recovery, slices were moved to standard ACSF (in mM: 125 NaCl, 2.5 KCl, 5 HEPES, 25 NaHCO₃, 1.25 NaH_2_PO₄, 12.5 D‐glucose, 2 CaCl_2_·2H_2_O [2 mL from 1 M stock], and 1 MgCl_2_·2H_2_O [1 mL from 1 M stock]) and maintained at room temperature for at least 45 min prior to recordings.

#### 2.5.2 Current‐Clamp Recordings

After incubation, coronal slices were transferred to the recording chamber of an upright microscope (Nikon FN1 Eclipse; Nikon Instruments, Melville, NY, USA), visualized using infrared differential interference contrast (IR‐DIC) optics at 40× magnification, and continuously perfused with oxygenated standard ACSF at room temperature.

Electrophysiological recordings were obtained using the Axopatch 200B amplifier which was connected to a Digidata 1550B digitizer (Molecular Devices, San Jose, CA, USA). Signals were sampled at 10 kHz and filtered at 5 kHz. Patch electrodes were made using thin‐walled borosilicate glass (1.5 mm OD × 1.17 mm ID × 100 mm; Harvard Apparatus, Holliston, MA, USA) using a two‐stage pull protocol on a P‐1000 puller (Sutter Instruments, Novato, CA, USA), yielding a tip resistance of 4–6 MΩ. Patch electrodes were filled with intracellular solution containing (in mM): 135 K‐gluconate, 4 KCl, 2 MgCl_2_, 10 HEPES, 0.3 EGTA, 4 Na_2_ATP, and 0.3 Na_2_GTP, adjusted to pH 7.3 with KOH. Membrane potentials were not corrected for the liquid junction potential, which was theoretically estimated to be approximately −15 mV.

After establishing whole‐cell access, neurons were held at −65 mV and allowed to stabilize for at least 3 min before data collection. Series resistance was monitored throughout the recording by applying a 10‐mV hyperpolarizing voltage step every minute and calculating Rs from the change in current. Recordings were excluded if series resistance exceeded 20 MΩ or changed by more than 20% during the experiment.

Frequency‐current (F‐I) relationships were obtained using 500 ms current injections ranging from −150 pA to +300 pA in 50 pA increments. Each step included a 100 ms pre‐step baseline and a 900 ms post‐step baseline, giving a total duration of 1500 ms per sweep. Inter‐step intervals were 1000 ms. Passive membrane properties (resting membrane potential [RMP], input resistance [RIN], membrane time constant, and capacitance) and active properties (action potential [AP] threshold, AP amplitude, AP halfwidth, afterhyperpolarization [AHP], and sag ratio) were quantified offline as described (Viteri, Bueschke, et al. 2025; Viteri, Kerr, et al. 2025). The difference between AP threshold and resting membrane potential (AP threshold—RMP) was also calculated for each neuron to determine the amount of depolarization required to reach AP threshold.

#### 2.5.3 Voltage‐Clamp Recordings

Following current‐clamp recordings, we targeted layer II/III of the primary motor cortex (atlas.brain‐map.org) for extracellular stimulation to assess synaptic inputs onto LVPNs. Layer II/III is a canonical source of feedforward drive to layer V in cortical circuits, providing both excitatory and inhibitory synaptic input onto LVPNs and representing the strongest known presynaptic connection to this layer (Weiler et al. 2008). Stimulating in layer II/III therefore allowed us to assess evoked synaptic responses onto LVPNs in young and aged mice. Layer II/III stimulation experiments were performed similarly to those previously reported (Coley and Gao 2019; Shao et al. 2023). A tungsten bipolar electrode (WE3ST30.1B10, Microprobes for Life Science, Gaithersburg, MD, USA) connected to an isolated pulse stimulator (Model 2100, A‐M Systems, Sequim, WA, USA) was lowered into layer II/III of the primary motor cortex. The stimulating electrode was positioned directly above the recorded LVPN within the same cortical column, with the electrode tip placed approximately 100–300 μm below the pial surface. This depth corresponds to layer II/III based on prior mouse motor cortex studies, which define layer II/III as approximately 100–300 μm below the pia or within the upper 8%–35% of total cortical depth, with layer V beginning deeper in the cortex at approximately 35% of total cortical depth (Swanson et al. 2021; Muñoz‐Castañeda et al. 2021; Commisso et al. 2018). Placement was also guided by the visible cortical layer boundaries described for mouse primary motor cortex, where layer organization has been mapped using soma depth and cytoarchitecture.

To isolate excitatory postsynaptic currents (EPSCs), LVPNs were voltage‐clamped at −70 mV (Cl^−^ reversal potential) to capture glutamatergic inward currents. Stimulation intensity was increased incrementally until EPSC amplitude saturated, defined as the point at which further increases in stimulation intensity no longer increased the evoked response. We refer to this throughout as the “maximum EPSC amplitude”. Amplitude was calculated as the difference between the baseline current and the peak inward current. Stimulation was then performed at 0.5 Hz for 3 min total. The first 2 min were used to allow the evoked response to stabilize, and only responses during the final minute were recorded and used for analysis. EPSC peak amplitude, halfwidth, and decay tau were averaged across all responses recorded during this final minute. PPR, a measure of short‐term synaptic plasticity (Jackman and Regehr 2017), was assessed by delivering two pulses at the same stimulation intensity with a 75 ms interpulse interval, repeated three times at 5‐s intervals. PPR was calculated as EPSC_2_/EPSC_1_ (Fleidervish and Gutnick 1995), averaged across sweeps.

Immediately following EPSC recordings, ACSF containing 20 mM tetraethylammonium (TEA), prepared by equimolar substitution of NaCl, was perfused for 10 min to reduce outward K^+^ currents (Viteri, Bueschke, et al. 2025; Viteri, Kerr, et al. 2025). To isolate IPSCs, LVPNs were held at 0 mV (glutamate reversal potential), and layer II/III was stimulated using the same approach described for EPSCs. Evoked IPSCs were recorded for 3 min total using the same stimulation protocol, with the first 2 min used for stabilization and only the final minute recorded and used for analysis. IPSC maximum amplitude, halfwidth, decay tau, and PPR were measured using the same procedures described for EPSCs. As with EPSCs, amplitude was calculated as the difference between the baseline current and the peak response; for IPSCs, this was measured as the peak outward current. To validate that the recorded EPSCs and IPSCs represented receptor‐mediated synaptic currents, separate pharmacological experiments were performed. 6,7‐dinitroquinoxaline‐2,3‐dione (DNQX; 20 μM; Sigma‐Aldrich, St. Louis, MO, USA) was applied to block AMPA (α‐amino‐3‐hydroxy‐5‐methyl‐4‐isoxazolepropionic acid) receptor‐mediated currents (D'amour and Froemke 2015), and bicuculline (50 μM; Sigma‐Aldrich, St. Louis, MO, USA) was applied to block GABA_A_ receptor‐mediated currents (Kapur et al. 1997). DNQX abolished the evoked EPSC, while bicuculline abolished the evoked IPSC, confirming that the recorded currents represented AMPA receptor‐mediated excitatory and γ‐aminobutyric acid type A (GABA_A_) receptor‐mediated inhibitory synaptic responses, respectively (Figure S3A).

### 2.6 Molecular Analysis

To investigate molecular markers associated with LVPN hyperexcitability, we quantified single‐cell mRNA expression of voltage‐gated ion channel subunits known to carry the persistent inward current (PIC) (Heckman et al. 2008)‐specifically the voltage‐gated sodium channel subunits Nav1.6 (*Scn8a*) and Nav1.1 (*Scn1a*), the voltage‐gated calcium channel subunit Cav1.3 (*Cacna1d*) ‐ as well as the serotonin receptor subtype 2C (*Htr2c*), which modulates the PIC. Transcript quantification was performed at the single‐cell level using a cell‐harvesting approach adapted from Pellizzari et al. (2023) with digital polymerase chain reaction (dPCR). Single‐cell transcript measurements were performed in a separate harvesting cohort (*N* = 4 mice and *n* = 20 cells per age group); no electrophysiological measurements were collected from those harvested cells.

#### 2.6.1 Cell Harvesting

Following identification of LVPNs as described above, glass pipettes were pre‐filled with 3.0–3.5 μL of internal electrode solution. After achieving the whole‐cell configuration, the membrane potential was held at −65 mV and the cell was allowed to stabilize for 3 min. A voltage‐clamp stepping protocol was then initiated in which the membrane was stepped from −5 to +20 mV at 5 ms intervals to increase electrophoretic attraction of negatively charged RNA molecules toward the positively charged pipette tip solution (Fuzik et al. 2016). Cytoplasmic and nuclear contents were aspirated over a structured 9‐min negative pressure protocol using a 60 mL syringe: 3 min of gentle steady negative pressure, followed by 3 min of moderate negative pressure, and a final 3 min of strong negative pressure. Successful aspiration of cytoplasmic and nuclear contents was confirmed visually by the characteristic shriveling of the LVPN soma, which typically became apparent by the 6‐min mark. Upon completion of the protocol, the electrode holder—with the glass pipette still attached—was carefully removed from the bath, and the pipette tip was firmly broken into the bottom of a 1.5 mL microcentrifuge tube containing 100 μL of RNA lysis buffer (Quick‐RNA MicroPrep Kit, Zymo Research, Irvine, California, USA), followed by a brief burst of positive pressure to expel the pipette contents. Tubes were vortexed for 3 s, briefly centrifuged, sealed with Parafilm to prevent evaporation, and stored at −80°C until processing.

#### 2.6.2 RNA Isolation and cDNA Synthesis

Total RNA was extracted from individual LVPNs using a column‐based extraction kit per the manufacturer's instructions (Quick‐RNA MicroPrep Kit, Zymo Research). Complementary DNA (cDNA) was synthesized from the total RNA of each single cell in a 14 μL reverse transcription reaction using SuperScript IV VILO reverse transcriptase per the manufacturer's instructions (Thermo Fisher Scientific, Waltham, Massachusetts, USA). To ensure sufficient template for downstream quantification, target transcripts—*Scn8a*, *Scn1a*, *Cacna1d*, and *Htr2c*—were pre‐amplified in a 25 μL reaction using TaqMan PreAmp Master Mix (Thermo Fisher Scientific) per the manufacturer's instructions. The pre‐amplified product was then diluted 7.5‐fold with nuclease‐free water (final volume: 187.5 μL) and used as the cDNA template for all subsequent dPCR reactions.

#### 2.6.3 dPCR

Gene expression was quantified by dPCR on a QuantStudio Absolute Q platform (Thermo Fisher Scientific). Pre‐designed, pre‐validated TaqMan primer/probe assays were used for each target, with assay IDs as follows: *Scn8a* (Mm00488110_m1), *Scn1a* (Mm00450580_m1), *Cacna1d* (Mm01209927_g1), and *Htr2c* (Mm00434127_m1). Assays were multiplexed within each dPCR reaction; all probe/fluorophore combinations were pre‐validated by the manufacturer (Thermo Fisher Scientific) to prevent cross‐reactivity. Each 10 μL reaction contained 2 μL of 5× Absolute Q DNA dPCR Master Mix, 0.5 μL of the corresponding primer/probe assay (0.5× final concentration), 1 μL of cDNA template, and nuclease‐free water to volume. Reactions were loaded onto MAP16 plates and thermocycled under the following conditions: initial polymerase activation at 96°C for 10 min, followed by 40 cycles of denaturation at 96°C for 5 s and combined annealing/extension at 60°C for 15 s. Absolute transcript copy number per microliter was quantified using QuantStudio Absolute Q Software v6.3 (Thermo Fisher Scientific).

Because total ribosomal rRNA (18S rRNA) constitutes approximately 80%–90% of total eukaryotic RNA, the 18S rRNA gene was used as a housekeeping reference to control for variability in the volume of cytoplasm aspirated during cell harvesting. Similar approaches using 18S rRNA normalization for transcript quantification from individually harvested or patched cells have been previously reported (Pellizzari et al. 2023; Jackson et al. 2024; Dvorak et al. 2023; Merrill et al. 2015). Copy number per microliter for each target transcript (*Scn8a*, *Scn1a*, *Cacna1d*, *Htr2c*) was normalized by dividing by the copy number per microliter of the mouse 18S rRNA housekeeping assay (Assay ID: Mm03928990_g1). Unnormalized values for each transcript are reported in Supporting Information, Figure S2. 18S rRNA copies/μL were not significantly different between groups (Figure S2).

### 2.7 Statistical Analysis

All statistical analyses were conducted in GraphPad Prism version 10.1.1 (GraphPad Software, San Diego, CA, USA) and R (version 4.3.1) via RStudio (version 2023.09.0 + 463). Datasets involving two factors, such as F‐I relationships, were analyzed using a two‐way ANOVA followed by Tukey's HSD test for post hoc multiple comparisons. Comparisons between only two groups were evaluated using unpaired Student's *t*‐tests. Data normality was assessed using the Shapiro–Wilk test. All results are reported as mean ± standard deviation (SD), and statistical significance was defined as *p* < 0.05.

#### 2.7.1 Principal Component Analysis

To characterize the overall separation between young and aged animals, a principal component analysis (PCA) was performed in R using all variables collected—both cortical (corticomuscular output, as well as LVPN intrinsic, active, passive, and synaptic properties) and neuromuscular/behavioral (motor behavior, in vivo electrophysiology, and muscle contractility) ‐ across all individual animals. Variables were z‐score standardized prior to PCA to equalize their contribution regardless of scale. Each data point in the PCA plot and correlation analysis (see below) represents a single animal. Because multiple LVPN patch clamp recordings were obtained per animal, cortical variables were averaged across all individual LVPN recordings from that animal to yield a single representative value per animal prior to analysis. The first two principal components (PC1 and PC2) were retained for visualization, and 95% confidence ellipses were plotted for each age group.

#### 2.7.2 Correlation Analysis

To determine the relationships between cortical variables and neuromuscular and behavioral outcome measures, we computed pairwise Pearson correlations between all cortical variables (*n* = 25) and all neuromuscular/behavioral variables (*n* = 10), yielding 250 unique correlation pairs. Cortical variables were averaged across all LVPN recordings from that animal to yield a single per‐animal value prior to analysis. Pearson correlations were computed across all animals combined (young and aged), rather than within each age group separately. This was intentional: our goal was to determine whether cortical physiological changes covaried with the broader neuromuscular and behavioral phenotype across the phenotypic range spanned by our young and aged cohorts, not to identify relationships that were independent of age within either age group. Within‐group correlations were also underpowered given sample sizes (*N* = 16 per group). To control for the elevated false discovery rate inherent to 250 simultaneous comparisons, *p*‐values were corrected using the Benjamini‐Hochberg false discovery rate (FDR) procedure. Only correlations surviving FDR correction (adjusted *p* < 0.05) were considered statistically significant and are reported. To further evaluate whether pooling was statistically appropriate, we tested whether each cortical—neuromuscular/behavioral relationship differed by age using an interaction model (cortical variable × age group) fit to the full sample, in both directions (neuromuscular/behavioral ~ cortical × age, and cortical ~ neuromuscular/behavioral × age), with the same FDR correction applied. No pair showed a significant age interaction in either direction (0 of 250 pairs, FDR‐corrected *p* < 0.05), indicating no evidence that the slope of these relationships differed between young and aged animals (Table S3). Additionally, uncorrected Pearson correlations were computed separately within young and aged animals and are provided in Supporting Information (Figure S4, Tables S1 and S2) for transparency.

#### 2.7.3 Identification of the Top Cortical Correlate of Neuromuscular Dysfunction

To identify which cortical variable was the strongest correlate of overall neuromuscular status, simple linear regressions were performed in R for each of the 25 cortical variables—averaged across all LVPN recordings per animal—against the first principal component score derived from a PCA of all neuromuscular and behavioral variables (neuromuscular PC1; hereafter referred to as the neuromuscular dysfunction index), which was used as a composite index of overall neuromuscular function (Nakazato et al. 2020; Boccia et al. 2026). The coefficient of determination (*R* ^2^) from each regression was extracted, and cortical variables were ranked by *R* ^2^ in descending order. As above, cortical variables represent per‐animal averages across all LVPN recordings.

#### 2.7.4 Mediation Analysis

To test whether cortical function statistically mediated the effect of aging on neuromuscular dysfunction, a mediation analysis was performed in R using the *mediation* package (Byeon and Lee 2023). Mediation analysis is a well‐established statistical framework used across biomedical and neuroscience research to test whether the relationship between an independent variable and an outcome is statistically accounted for by an intermediate variable (Baron and Kenny 1986). Rather than simply establishing that two variables are associated, mediation analysis asks a more specific question: *is the relationship between* variable A *and* variable C *statistically accounted for* by *an intermediate* variable B? It decomposes the total effect of an independent variable (here, age) on an outcome (here, the neuromuscular dysfunction index—a composite score of neuromuscular function derived from PC1 of all neuromuscular and behavioral variables; see above) into: (1) a *direct effect*—the portion of the age effect on neuromuscular dysfunction that remains after accounting for the mediator; and (2) an *indirect effect*—the portion of the total effect statistically accounted for by the mediator. This framework therefore allows one to determine not just *whether* aging and neuromuscular dysfunction are associated, but *whether their relationship is statistically explained through an intermediate variable*.

The analysis tested a proposed model in which aging (young vs. aged, binary independent variable) is associated with the neuromuscular dysfunction index (outcome) both directly and indirectly through LVPN firing frequency at 50 pA (averaged across all LVPN recordings per animal), which was identified as the top cortical correlate of the neuromuscular dysfunction index (Figure 6B) and used as the mediator in this a priori model. The directionality of this model reflects a working hypothesis rather than a direction established by the data, given the observational nature of these measurements; we therefore also performed the analysis with the mediator and outcome reversed, described in the next paragraph. The first mediation model was structured as follows: (a) path *a* ➔ a linear regression of LVPN firing frequency at 50 pA on age, quantifying how strongly aging is associated with the mediator; (b) path *b* ➔ a linear regression of the neuromuscular dysfunction index on both LVPN firing frequency at 50 pA and age simultaneously, quantifying how strongly the mediator is associated with neuromuscular dysfunction while controlling for age; and (c) the direct effect *c'* ➔ the residual effect of age on the neuromuscular dysfunction index after statistically controlling for the mediator. The average causal mediation effect (ACME)– the indirect effect of aging on the neuromuscular dysfunction index operating specifically through LVPN firing frequency—was computed as the product of paths *a* × *b* (Baron and Kenny 1986).

Statistical significance of the ACME was assessed using 10,000 bootstrapped resamples with bias‐corrected 95% confidence intervals (Preacher and Hayes 2008). The proportion mediated was calculated as ACME divided by the total effect of age on the neuromuscular dysfunction index, reflecting how much of the age‐related effect on neuromuscular dysfunction is statistically accounted for by the pathway through LVPN hyperexcitability.

Because the directionality of this model cannot be established from observational data alone, we additionally performed the mediation analysis with the mediator and outcome reversed: age as the independent variable, the neuromuscular dysfunction index as the mediator, and LVPN firing frequency at 50 pA as the outcome, using the same procedure described above. Both models are reported. These observational models do not establish causal direction or a biological mechanism and may be affected by unmeasured mediator–outcome confounding.

## 3 Results

### 3.1 Aged Mice Are Weak and Exhibit Impairments in Neuromuscular Excitability and Contractile Function

We first assessed motor behavior (Figure 1A–C). All‐limb grip strength (5.46 ± 1.05 g/g BW vs. 9.76 ± 1.31 g/g BW; *p* < 0.0001), rotarod latency to fall (12.12 ± 3.17 s vs. 15.37 ± 3.59 s; *p* = 0.0109), and weighted cart pull (9.71 ± 2.33 g/g BW vs. 16.31 ± 1.66 g/g BW; *p* < 0.0001) were all reduced in aged mice compared to young. Raw non‐normalized values for grip strength and the weighted cart pull exhibited the same age‐related reductions (Figure S1).

We next assessed neuromuscular excitability (Figure 1D–H). CMAP amplitude was reduced in aged mice (75.62 ± 17.05 mV vs. 92.72 ± 9.20 mV; *p* = 0.0018) while SMUP amplitude was increased in aged mice (202.9 ± 24.01 μV vs. 178.3 ± 25.76 μV; *p* = 0.0091). MUNE was reduced in aged mice (368.4 ± 80.0 units vs. 470.3 ± 71.7 units; *p* = 0.0007). RNS % decrement at 50 Hz was increased in aged mice (−36.94% ± 8.95% vs. −22.38% ± 6.80%; *p* < 0.0001). Representative CMAP traces illustrate these differences (Figure 1D).

We also assessed muscular contractility (Figure 1I–K). Both twitch (0.071 ± 0.019 mN‐m/g BW vs. 0.094 ± 0.011 mN‐m/g BW; *p* = 0.0003) and tetanic torque (0.307 ± 0.056 mN‐m/g BW vs. 0.471 ± 0.053 mN‐m/g BW; *p* < 0.0001) were reduced in aged mice compared to young. Representative twitch and tetanic traces illustrate these differences (Figure 1I).

Together, these results show that aged mice exhibit impairments in motor behavior, neuromuscular excitability, and contractile function.

### 3.2 Aged Mice Exhibit Enhanced Cortical Output to Muscle

We next measured cranial and cervical MEPs to assess cortical and spinal output to muscle, respectively (Figure 2A shows the respective stimulation configurations). Cervical MEP amplitude was reduced in aged mice compared to young (0.045 ± 0.013 mV/CMAP vs. 0.103 ± 0.044 mV/CMAP; *p* < 0.0001), while cranial MEP amplitude was increased (0.020 ± 0.012 mV/CMAP vs. 0.007 ± 0.005 mV/CMAP; *p* = 0.0009). Non‐normalized raw MEP values showed the same age‐related trends: cervical MEP amplitude was reduced in aged mice (3.427 ± 1.321 mV vs. 8.434 ± 2.690 mV; *p* < 0.0001), while cranial MEP amplitude was increased (1.544 ± 1.024 mV vs. 0.673 ± 0.439 mV; *p* = 0.0052) (Figure 2B,C). Representative traces show the reduction in cervical MEP amplitude and the increase in cranial MEP amplitude in aged mice (Figure 2B,C).

Together, these results show reduced spinal output to muscle, but increased cortical output to muscle in aged mice.

### 3.3 Aged Layer V Neurons Are Hyperexcitable and Have Altered Passive Properties

**FIGURE 3:**
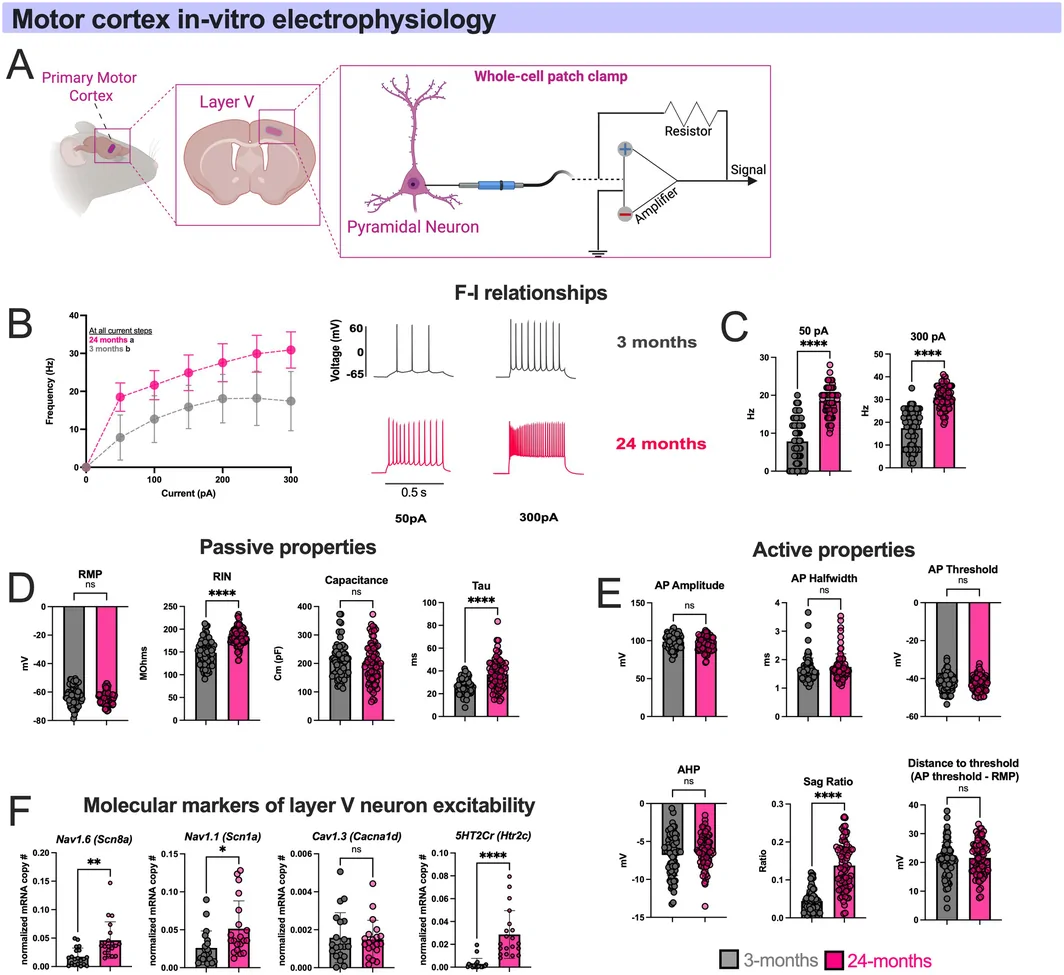
Aged LVPNs are hyperexcitable and exhibit upregulated molecular markers of excitability. (A) Schematic of whole‐cell patch clamp recordings from LVPNs of the primary motor cortex. Whole‐cell recordings were obtained from *N* = 16 mice per group (young: *n* = 77 LVPN recordings; aged: *n* = 83 LVPN recordings). (B, C) Firing frequency across 50–300 pA was higher in aged mice at all current steps, with representative traces shown at 50 and 300 pA. (D) Among passive membrane properties, R_IN_ and the time constant (tau) were increased in aged mice, while the RMP and capacitance were unchanged. (E) No active membrane properties differed between groups with the exception of sag ratio, which was increased in aged mice. (F) 18S‐normalized transcript values for the PIC‐related genes Nav1.6 Nav1.6 (*Scn8a*), Nav1.1 (*Scn1a*), and for 5‐HT2C receptor (*Htr2c*) were all increased in single LVPNs from aged mice, while Cav1.3 (*Cacna1d*) was unchanged. All target transcript measurements were normalized to 18S rRNA. Molecular analysis was performed in a separate cohort (*N* = 4 mice per group; *n* = 20 cells per group). Significance: *p < 0.05, **p < 0.01, ****p < 0.0001. Data are presented as means ± SD. *Figure made using BioRender*.

To determine whether the enhanced cortical motor output observed in vivo reflected changes in intrinsic properties of LVPNs, we performed whole‐cell patch clamp recordings in the primary motor cortex (Figure 3A). Firing frequency of LVPNs across the full F‐I relationship (Figure 3B) was significantly higher in aged mice compared to young at all current steps from 50 to 300 pA (two‐way ANOVA: age effect *F*(1, 936) = 838.3, *p* < 0.0001; current effect *F*(5, 936) = 96.70, *p* < 0.0001; interaction *F*(5, 936) = 4.059, *p* = 0.0012), with post hoc comparisons confirming significant differences at these current steps (all *p* < 0.0001). Firing frequency was increased in aged mice at both 50 pA (18.48 ± 3.74 Hz vs. 7.84 ± 5.95 Hz; *p* < 0.0001) and 300 pA (30.90 ± 4.77 Hz vs. 17.43 ± 7.81 Hz; *p* < 0.0001). Representative traces illustrate the increase in firing frequency in aged mice at both current steps (Figure 3B).

Among passive membrane properties (Figure 3D), *R*_IN_ (184.2 ± 19.2 MΩ vs. 148.3 ± 27.9 MΩ; *p* < 0.0001) and the time constant (tau) (37.02 ± 13.21 ms vs. 26.37 ± 6.43 ms; *p* < 0.0001) were increased in aged mice compared to young. The RMP (−64.30 ± 4.08 mV vs. −63.25 ± 6.46 mV; *p* = 0.2271) and capacitance (202.8 ± 70.0 pF vs. 209.2 ± 58.0 pF; *p* = 0.5331) were not different between groups.

Among active membrane properties (Figure 3E), AP amplitude (96.48 ± 8.95 mV vs. 98.75 ± 8.64 mV; *p* = 0.1080), AP halfwidth (1.74 ± 0.46 ms vs. 1.64 ± 0.41 ms; *p* = 0.1534), AP threshold (−42.72 ± 3.97 mV vs. −42.37 ± 4.66 mV; *p* = 0.6178), distance to threshold (AP threshold − RMP) (21.58 ± 5.85 mV vs. 20.88 ± 6.07 mV; *p* = 0.4581), and AHP (−6.28 ± 2.17 mV vs. −6.79 ± 2.73 mV; *p* = 0.2000) were not different between groups. Sag ratio was increased in aged mice (0.138 ± 0.063 vs. 0.044 ± 0.029; *p* < 0.0001).

Together, these results show that LVPNs of the primary motor cortex are hyperexcitable in aged mice, consistent with the findings of Viteri et al. (Viteri, Bueschke, et al. 2025).

### 3.4 Aged Layer V Neurons Exhibit Upregulated Molecular Markers of Neuronal Excitability

Given our prior findings that the persistent inward current (PIC), a powerful amplifier of neuronal excitability and repetitive firing (Heckman et al. 2008), is increased in aged LVPNs (Viteri, Bueschke, et al. 2025), we assessed whether transcriptional markers of PIC‐mediated excitability were also altered in this population. mRNA was harvested from individually patched neurons, and we targeted transcripts encoding the primary PIC channel subunits Nav1.6 (Scn8a), Nav1.1 (Scn1a), and Cav1.3 (Cacna1d), as well as the 5‐HT2C receptor (Htr2c), a known modulator of PIC function (Figure 3F).

Nav1.6 (*Scn8a*) (0.046 ± 0.032 vs. 0.017 ± 0.015; *p* = 0.0011) and Nav1.1 (*Scn1a*) (0.051 ± 0.037 vs. 0.026 ± 0.022; *p* = 0.0126) 18S‐normalized transcript values were both increased in aged mice compared to young. Cav1.3 (*Cacna1d*) was not different between groups (0.0015 ± 0.0010 vs. 0.0016 ± 0.0013; *p* = 0.8067). 5‐HT2C receptor (*Htr2c*) transcript values were also increased in aged mice (0.029 ± 0.021 vs. 0.003 ± 0.005; *p* < 0.0001). Raw non‐normalized values showed the same overall trend; however, Nav1.6 (*Scn8a*) did not reach statistical significance, although it trended higher in aged mice (Figure S2).

Together, these results show that PIC‐related transcripts were differentially expressed in aged LVPNs, providing candidate molecular correlates associated with the hyperexcitable phenotype. Specifically, the two genes encoding the sodium channel subunits of the PIC, Nav1.6 (*Scn8a*) and Nav1.1 (*Scn1a*), were upregulated in aged neurons, consistent with the direction of the sodium PIC increase we reported previously (Viteri, Bueschke, et al. 2025).

### 3.5 Aged LVPNs Exhibit Altered Excitatory and Inhibitory Synaptic Responses Following Layer II/III Stimulation

**FIGURE 4:**
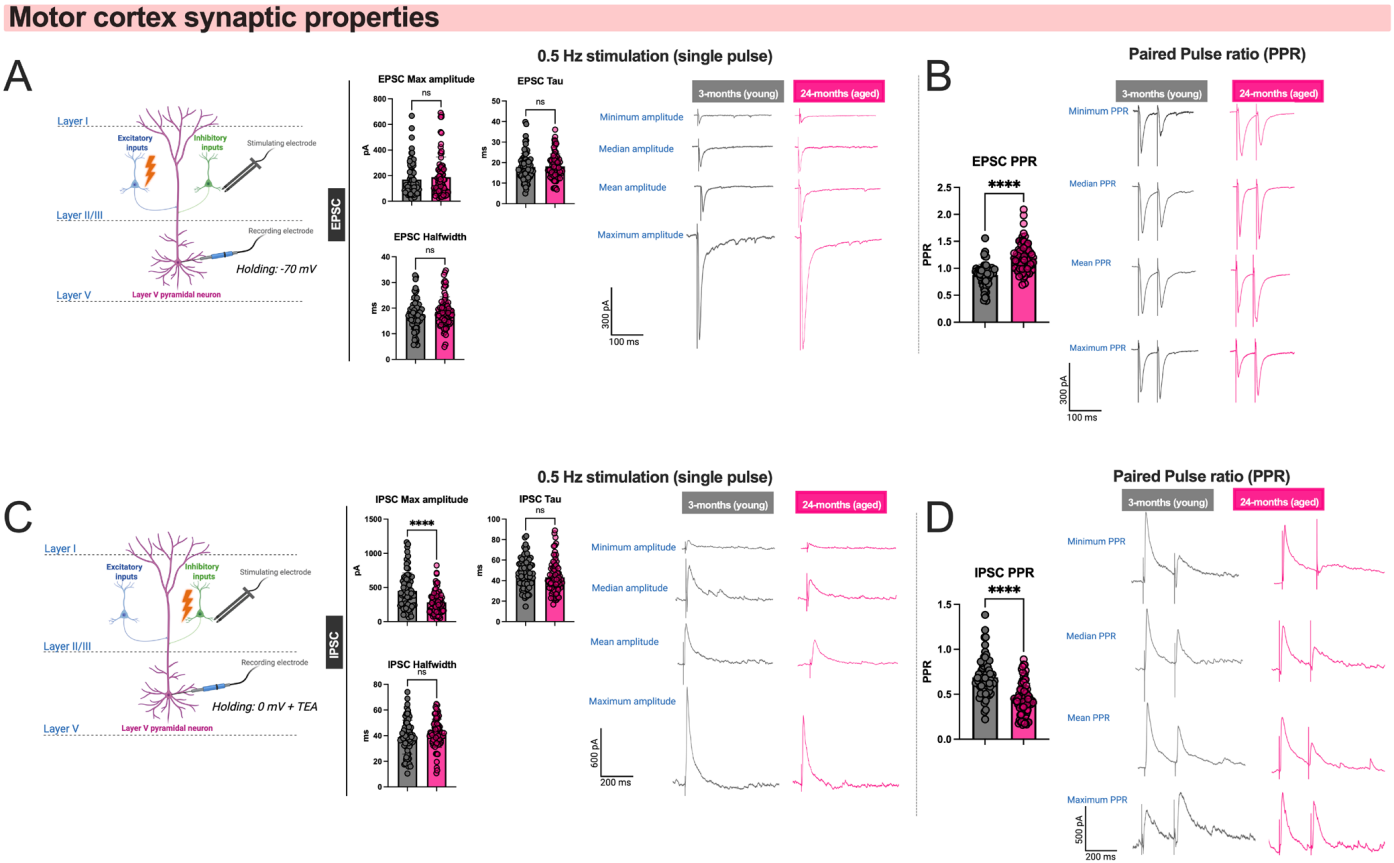
Aged LVPNs exhibit altered excitatory and inhibitory synaptic responses following layer II/III stimulation. (A) Schematic of excitatory synaptic input recordings from LVPNs held at −70 mV. EPSC maximum amplitude, tau, and halfwidth were unchanged between groups under 0.5 Hz stimulation. Representative traces show the minimum, median, mean, and maximum EPSC amplitudes recorded in each age group. (B) EPSC PPR was increased in aged mice compared to young. Representative paired‐pulse traces show minimum, median, mean, and maximum PPR values for each age group. (C) Schematic of inhibitory synaptic input recordings from LVPNs held at 0 mV in the presence of TEA. IPSC maximum amplitude was reduced in aged mice, while tau and halfwidth were unchanged under 0.5 Hz stimulation. Representative traces show the minimum, median, mean, and maximum IPSC amplitudes recorded in each age group. (D) IPSC PPR was decreased in aged mice compared to young. Representative paired‐pulse traces show minimum, median, mean, and maximum PPR values for each age group. Synaptic recordings were obtained from *N* = 16 mice/group (young *n* = 77 LVPN recordings, aged *n* = 83 LVPN recordings). Significance: ****p < 0.0001. Data are presented as means ± SD. *Figure made using BioRender*.

To assess whether changes in synaptic inputs onto LVPNs could contribute to their hyperexcitability, we examined both excitatory and inhibitory synaptic currents (Figure 4). Under 0.5 Hz stimulation, maximum EPSC amplitude (188.0 ± 157.9 pA, median 141.2 pA, variance 24,932 pA^2^ vs. 169.9 ± 128.4 pA, median 126.0 pA, variance 16,487 pA^2^; *p* = 0.4278), tau (18.19 ± 6.10 ms, median 16.98 ms, variance 37.3 ms^2^ vs. 17.87 ± 6.65 ms, median 16.38 ms, variance 44.2 ms^2^; *p* = 0.7585), and halfwidth (18.55 ± 5.84 ms, median 17.66 ms, variance 34.1 ms^2^ vs. 17.45 ± 5.75 ms, median 16.93 ms, variance 33.0 ms^2^; *p* = 0.2362) were all unchanged (Figure 4A), indicating that the measured maximum EPSC amplitude and kinetics did not differ between young and aged mice. Representative EPSC traces spanning the minimum, median, mean, and maximum amplitude values demonstrate the range of evoked responses recorded in each age group (Figure 4A). The full distribution of EPSC amplitudes across all recordings is shown in Figure S3B. Interestingly, EPSC PPR was increased in aged mice compared to young (1.198 ± 0.260, median 1.164, variance 0.0676 vs. 0.874 ± 0.200, median 0.898, variance 0.0398; *p* < 0.0001; Figure 4B), indicative of synaptic facilitation and reduced initial presynaptic release probability at excitatory inputs onto LVPNs. Representative EPSC traces spanning the minimum, median, mean, and maximum PPR values illustrate the change in PPR in aged mice (Figure 4B).

IPSC maximum amplitude was reduced in aged mice under 0.5 Hz stimulation (283.3 ± 163.6 pA, median 260.0 pA, variance 26,765 pA^2^ vs. 450.5 ± 262.7 pA, median 422.5 pA, variance 69,011 pA^2^; *p* < 0.0001), while tau (43.27 ± 15.70 ms, median 40.85 ms, variance 246.5 ms^2^ vs. 47.88 ± 14.29 ms, median 46.26 ms, variance 204.2 ms^2^; *p* = 0.0516) and halfwidth (41.87 ± 11.06 ms, median 41.80 ms, variance 122.3 ms^2^ vs. 38.86 ± 13.20 ms, median 38.32 ms, variance 174.2 ms^2^; *p* = 0.1272) were unchanged (Figure 4C). Representative IPSC traces spanning the minimum, median, mean, and maximum amplitude values demonstrate the range of evoked responses recorded in each age group (Figure 4C). The full distribution of IPSC amplitudes across all recordings is shown in Figure S3B. Interestingly, IPSC PPR was also decreased in aged mice (0.446 ± 0.176, median 0.437, variance 0.0309 vs. 0.688 ± 0.202, median 0.653, variance 0.0406; *p* < 0.0001; Figure 4D), indicative of synaptic depression and increased initial presynaptic release probability at inhibitory inputs onto LVPNs. Representative IPSC traces spanning the minimum, median, mean, and maximum PPR values illustrate the changes in PPR in aged mice (Figure 4D).

Together, these results show age‐related changes in excitatory and inhibitory synaptic responses evoked by layer II/III stimulation, with increased EPSC PPR, reduced IPSC amplitude, and decreased IPSC PPR in aged LVPNs.

### 3.6 Primary Motor Cortex LVPN Hyperexcitability and Altered Motor Cortex Synaptic Dynamics Strongly Correlate With Neuromuscular and Behavioral Dysfunction in Aged Mice

**FIGURE 5:**
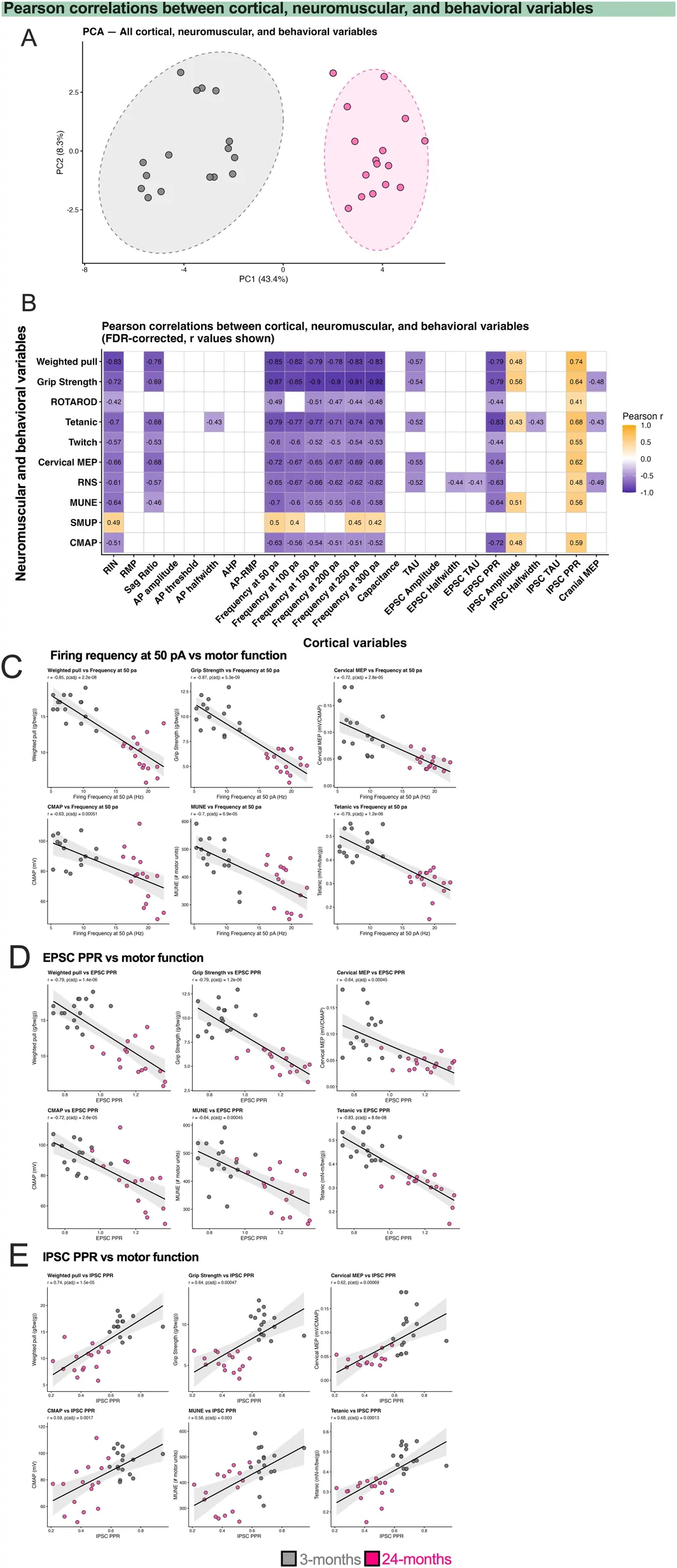
Primary motor cortex LVPN hyperexcitability and altered motor cortex synaptic dynamics strongly correlate with neuromuscular and behavioral dysfunction in aged mice. (A) PCA of all 35 cortical, neuromuscular, and behavioral variables (25 cortical, 10 neuromuscular/behavioral) measured from the same animals. PC1 (43.4%) and PC2 (8.3%) are shown. Shaded ellipses represent 95% confidence intervals for each age group, showing clear separation between young and aged mice in PC space. (B) Heatmap of significant Pearson correlations between cortical, neuromuscular, and behavioral variables computed across both age groups combined to maximize variance across the full young‐to‐aged phenotypic range. Only correlations meeting FDR‐corrected *p* < 0.05 are displayed. Of the 250 total pairwise correlations, 109 were significant, with the large majority being negative (*n* = 90) and a smaller subset being positive (*n* = 19). Purple cells indicate negative correlations; yellow cells indicate positive correlations. (C) Representative scatter plots of firing frequency at 50 pA against six neuromuscular and behavioral variables. Negative correlations indicate that higher LVPN firing frequency is associated with worse neuromuscular and behavioral outcomes. (D) Representative scatter plots of EPSC PPR against six neuromuscular and behavioral variables, showing the same negative pattern as firing frequency. (E) Representative scatter plots of IPSC PPR against six neuromuscular and behavioral variables, where lower IPSC PPR is associated with worse neuromuscular and behavioral outcomes. For all scatter plots, correlations were computed across both age groups combined; young and aged animals are shown in separate colors for qualitative visualization of group separation. Shaded regions represent 95% confidence intervals of the regression line. *r* and *p*(adj) values are shown for each plot. Significance threshold: FDR‐corrected *p* < 0.05.

To examine the relationship between cortical, neuromuscular, and behavioral outcomes measured from the same animals, we first applied PCA to all 35 cortical, neuromuscular, and behavioral variables (25 cortical, 10 neuromuscular/behavioral) to visualize the overall multivariate structure of the data (Figure 5A). PC1 explained 43.4% and PC2 explained 8.3% of total variance, with 95% confidence ellipses showing clear separation between young and aged mice along PC1, indicating that the combined cortical, neuromuscular, and behavioral profile reliably distinguishes the two age groups at a multivariate level.

We then performed Pearson correlations between all 25 cortical variables and 10 neuromuscular and behavioral variables measured from the same animals, yielding 250 pairwise correlations computed across both age groups combined to characterize covariation across the full young‐to‐aged phenotypic range (Figure 5B). Only correlations meeting an FDR‐corrected threshold of *p* < 0.05 are displayed in the heatmap. Of the 250 correlations, 109 were significant, the large majority of which were negative (*n* = 90), with a smaller subset being positive (*n* = 19). The cortical variables firing frequency, R_IN_, sag ratio, EPSC PPR, IPSC amplitude, IPSC PPR, and cranial MEP showed the broadest pattern of significant correlations with neuromuscular and behavioral variables, while active properties (amplitude, halfwidth, threshold, AHP, and AP‐RMP threshold difference), capacitance, and EPSC kinetics were largely uncorrelated with these outcomes.

Representative scatter plots illustrating the direction and magnitude of key correlations between cortical variables and neuromuscular and behavioral variables are shown in Figure 5C–E. All scatter plots display both young and aged animals combined, to denote the full young‐to‐aged phenotypic range; young and aged animals are shown in separate colors for qualitative visualization of group separation. Shaded regions represent 95% confidence intervals of the regression line. Representative correlations are shown for three cortical variables—firing frequency at 50 pA, EPSC PPR, and IPSC PPR—each plotted against six neuromuscular and behavioral variables spanning motor behavior, spinal output to muscle, neuromuscular excitability, and contractile function.

Firing frequency at 50 pA showed strong negative correlations with weighted cart pull (*r* = −0.85, *p*(adj) = 2.2e‐08), grip strength (*r* = −0.87, *p*(adj) = 5.3e‐09), cervical MEP (*r* = −0.72, *p*(adj) = 2.8e‐05), CMAP (*r* = −0.63, *p*(adj) = 5.1e‐04), MUNE (*r* = −0.70, *p*(adj) = 6.9e‐05), and tetanic torque (*r* = −0.79, *p*(adj) = 1.2e‐06; Figure 5C). Higher LVPN firing frequency was consistently associated with worse neuromuscular and behavioral outcomes across all measures.

EPSC PPR showed a similarly negative pattern with weighted cart pull (*r* = −0.79, *p*(adj) = 1.4e‐06), grip strength (*r* = −0.79, *p*(adj) = 1.2e‐06), cervical MEP (*r* = −0.64, *p*(adj) = 4.5e‐04), CMAP (*r* = −0.72, *p*(adj) = 2.8e‐05), MUNE (*r* = −0.64, *p*(adj) = 4.5e‐04), and tetanic torque (*r* = −0.83, *p*(adj) = 8.6e‐08; Figure 5D), consistent with the pattern observed for firing frequency.

IPSC PPR showed the opposite pattern, with positive correlations with weighted cart pull (*r* = 0.74, *p*(adj) = 1.5e‐05), grip strength (*r* = 0.64, *p*(adj) = 4.7e‐04), cervical MEP (*r* = 0.62, *p*(adj) = 6.9e‐04), CMAP (*r* = 0.59, *p*(adj) = 1.7e‐03), MUNE (*r* = 0.56, *p*(adj) = 3.0e‐03), and tetanic torque (*r* = 0.68, *p*(adj) = 1.3e‐04; Figure 5E). Lower IPSC PPR was associated with worse neuromuscular and behavioral outcomes.

Together, these results show that LVPN hyperexcitability and layer II/III‐evoked synaptic measures from the same animals are strongly and consistently correlated with worse motor, spinal, neuromuscular, and contractile outcomes, with excitatory variables showing negative and inhibitory variables showing positive correlations across these measures.

### 3.7 LVPN Firing Frequency Is the Strongest Correlate of Neuromuscular Dysfunction and Statistically Accounts for 75% of the Effect of Aging

**FIGURE 6:**
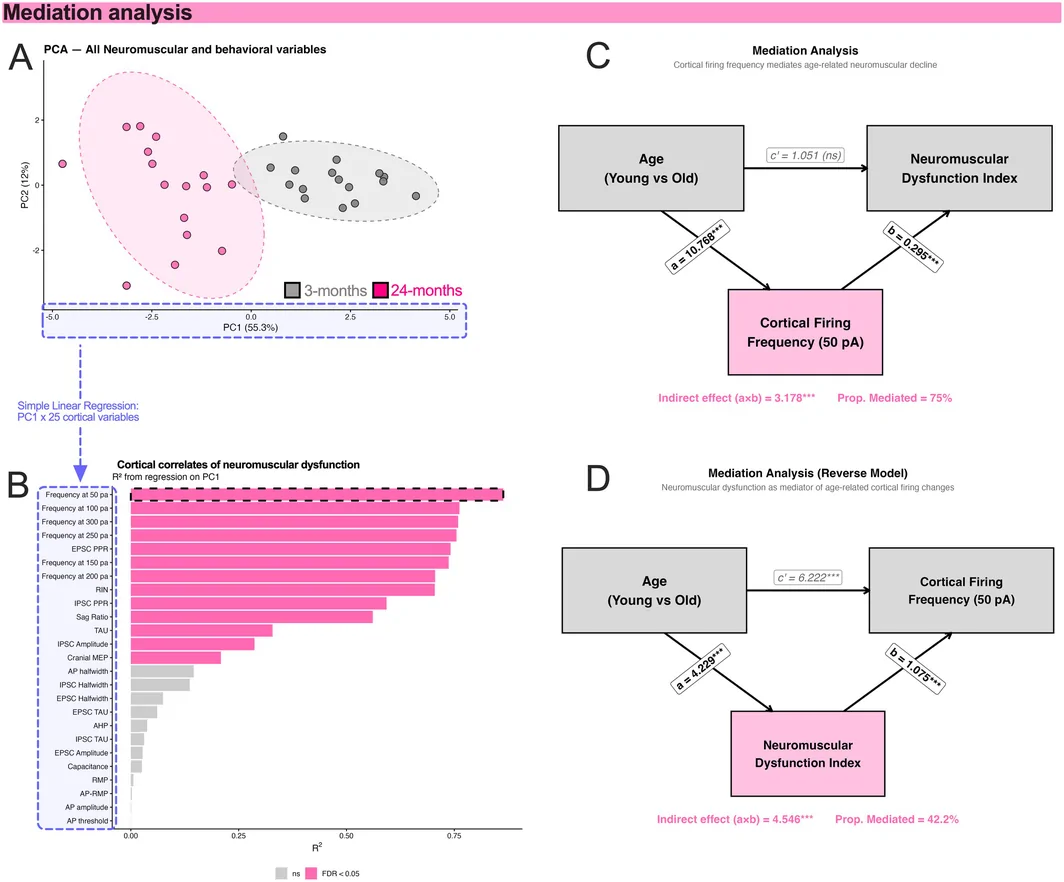
LVPN firing frequency is the strongest correlate of neuromuscular dysfunction and statistically accounts for 75% of the effect of aging. (A) PCA of all neuromuscular and behavioral variables. PC1 (55.3%) separated young and aged mice and was used as a composite neuromuscular dysfunction index for subsequent regression analysis. (B) Ranked bar plot of *R* ^2^ values from simple linear regressions of each of the 25 cortical variables against the neuromuscular dysfunction index. Bars are colored by significance status after FDR correction. Firing frequency at 50 pA was the single strongest correlate (dashed bar). (C) Mediation analysis showing that LVPN firing frequency at 50 pA statistically accounted for 75% of the effect of aging on the neuromuscular dysfunction index. Path coefficients and significance levels are shown. The direct effect (c') was non‐significant, while the indirect effect (ACME) through LVPN firing frequency was significant. (D) Reverse mediation model showing the neuromuscular dysfunction index as mediator and LVPN firing frequency at 50 pA as outcome. Unlike (C), the direct effect (c') remained significant, and a smaller proportion of the total effect was statistically accounted for by the mediator (42.2%).

Given the correlations between cortical and neuromuscular changes with age, we wanted to identify which cortical variable was most strongly correlated with overall neuromuscular dysfunction. We screened all cortical variables: simple linear regressions were performed for each of the 25 cortical variables against the neuromuscular dysfunction index—a composite score derived from PC1 of a PCA of all neuromuscular and behavioral variables (Figure 6A)—and cortical variables were ranked by *R* ^2^ in descending order (Figure 6B). Firing frequency at 50 pA emerged as the single strongest cortical correlate of the neuromuscular dysfunction index (*R* ^2^ = 0.90, FDR‐corrected *p* < 0.05).

While the regression analysis identified firing frequency at 50 pA as the top cortical correlate of the neuromuscular dysfunction index, it did not describe how much of the effect of aging on neuromuscular dysfunction was statistically explained by LVPN firing. To address this, we performed a mediation analysis—a statistical approach that tests how much of the effect of one variable on another is explained by a third intermediate variable—with age as the independent variable, LVPN firing frequency at 50 pA as the mediator, and the neuromuscular dysfunction index as the outcome (Figure 6C).

Aging was strongly correlated with LVPN firing frequency (path a = 10.768, *p* < 0.001), and LVPN firing frequency in turn was associated with the neuromuscular dysfunction index while controlling for age (path b = 0.295, *p* < 0.001). The direct effect of age on the neuromuscular dysfunction index after controlling for firing frequency was non‐significant (path c' = 1.051, 95% CI: −0.088–2.347, *p* = 0.072), while the indirect effect through LVPN firing frequency was significant (ACME = 3.178, 95% CI: 1.916–4.507, *p* < 0.001). The total effect of age on the neuromuscular dysfunction index was 4.229 (95% CI: 3.581–4.915, *p* < 0.001), with LVPN firing frequency statistically accounting for 75% of this effect (95% CI: 45.8%–102.2%).

Because these are observational data, the direction of this mediation model reflects an a priori assumption rather than a direction established by the data. We therefore also performed the analysis with the mediator and outcome reversed: age as the independent variable, the neuromuscular dysfunction index as the mediator, and LVPN firing frequency at 50 pA as the outcome (Figure 6D). In this reversed model, aging was also strongly associated with the neuromuscular dysfunction index (path a = 4.229, *p* < 0.001), and the neuromuscular dysfunction index was associated with LVPN firing frequency while controlling for age (path b = 1.075, *p* < 0.001). Unlike the original model, the direct effect of age on LVPN firing frequency remained significant after accounting for the mediator (path c' = 6.222, 95% CI: 3.235–8.861, *p* < 0.001), and the indirect effect through the neuromuscular dysfunction index was also significant (ACME = 4.546, 95% CI: 2.331–7.318, *p* < 0.001). The total effect of age on LVPN firing frequency was 10.768 (95% CI: 9.466–12.033, *p* < 0.001), with the neuromuscular dysfunction index accounting for 42.2% of this effect (95% CI: 21.0%–68.8%). Both models are presented as a statistical decomposition of variance; neither establishes which variable causally precedes the other.

Together, these results identify LVPN firing frequency at 50 pA as the single strongest correlate of the neuromuscular dysfunction index. Statistical mediation models tested in both directions show that LVPN firing frequency statistically accounted for 75% of the association between age and neuromuscular dysfunction, while neuromuscular dysfunction accounted for 42.2% of the association between age and LVPN firing frequency, though these observational data cannot establish the direction of this relationship.

## 4 Discussion

In this study, we showed that enhanced cortical output to muscle and primary motor cortex LVPN hyperexcitability are both strongly correlated with weakness and neuromuscular dysfunction in aged mice. We identified multiple potential mechanisms underlying these cortical changes, including altered excitatory and inhibitory synaptic responses evoked by layer II/III stimulation in the motor cortex (Figure 4) and upregulated molecular markers of excitability within single neurons of the aged motor cortex (Figure 3). Strikingly, correlation analysis across 250 cortical–neuromuscular/behavioral pairwise relationships from the same animals revealed that cortical excitability and synaptic properties are broadly and consistently correlated with neuromuscular and behavioral outcomes (Figure 5)—with firing frequency of layer V neurons emerging as the single strongest correlate of neuromuscular dysfunction and statistically accounting for 75% of the age effect on neuromuscular function, while the reverse analysis showed that neuromuscular dysfunction accounted for 42.2% of the age effect on LVPN firing frequency (Figure 6).

Our understanding of the neural mechanisms driving age‐related weakness has traditionally focused on changes at the level of the muscle, neuromuscular junction, and spinal cord. However, mounting evidence suggests that motor circuits in the brain are impaired in OAs, including altered cortical motor output to muscle and disrupted E/I balance, which may be direct contributors to age‐related weakness (Clark 2023). Yet, a common limitation across these studies has been the inability to identify the specific cell types and mechanisms underlying these changes, leaving the cortical basis of age‐related motor dysfunction largely unresolved.

Recently, we identified LVPNs of the primary motor cortex—a neuronal population that includes corticospinal neurons that transmit motor commands to spinal circuits (Grimsley et al. 2013)—as hyperexcitable in aged mice (Viteri, Bueschke, et al. 2025). An outstanding question, however, was whether this neuronal phenotype was associated with weakness and neuromuscular dysfunction, and to what extent. Here, we showed that aged mice exhibited reductions in neuromuscular excitability, spinal output to muscle, and muscle contractility (Figures 1 and 2), while cranial MEPs demonstrated enhanced cortical motor output to muscle in vivo (Figure 2). Patch‐clamp recordings from the same animals replicated our previous finding of LVPN hyperexcitability (Viteri, Bueschke, et al. 2025)–with increased firing frequency at all tested depolarizing steps from 50 to 300 pA (Figure 3). Critically, this enhanced cortical motor output was accompanied by intrinsic hyperexcitability of the constituent cortical neurons themselves. LVPNs also exhibited elevated membrane resistance (Figure 3), which reduces the current required to reach action potential threshold and increases neuronal excitability—a finding consistent with our previous observations in aged LVPNs (Viteri, Bueschke, et al. 2025), and with membrane resistance changes reported in other neurodegenerative contexts (Chen et al. 2025). The increased sag ratio detected in aged LVPNs (Figure 3) may reflect altered I_h_, a hyperpolarization‐activated depolarizing current whose dysregulation has similarly been linked to altered excitability across multiple neurological diseases (Chen et al. 2025; Combe and Gasparini 2021).

Our single‐neuron dPCR results further identified transcriptional changes in excitability‐related genes associated with LVPN hyperexcitability. Nav1.6 (*Scn8a*) and Nav1.1 (*Scn1a*)—two of the three sodium channel subunits contributing to the PIC, a powerful amplifier of neuronal excitability and repetitive firing (Heckman et al. 2008)—were elevated in aged LVPNs (Figure 3), consistent with the direction of the increased PIC previously identified electrophysiologically in aged LVPNs (Viteri, Bueschke, et al. 2025). Elevated *Htr2c* transcript levels in aged LVPNs further suggested a role for altered serotonergic signaling via the 5‐HT2C receptor, a known modulator of the PIC (Kerr et al. 2025; Heckman et al. 2008; D'Amico et al. 2013)—as a potential mechanism of LVPN hyperexcitability (Figure 3). The relevance of both is emphasized by evidence that their dysregulation is thought to contribute to motor dysfunction across sarcopenia (Kerr et al. 2025; Orssatto et al. 2023), spinal cord injury (D'Amico et al. 2013; ElBasiouny et al. 2010), and ALS (Viteri, Kerr, et al. 2025; Quinlan et al. 2011). Together, these results provide the first evidence of age‐associated transcriptional changes in excitability‐related genes within single neurons of the aged motor cortex, offering candidate molecular markers of LVPN hyperexcitability that may be linked to neuromuscular dysfunction. This is further supported by evidence that genetic variants associated with grip strength are significantly enriched in central nervous system (CNS) regulatory regions, with expression of several brain transcripts associated with greater grip strength (Willems et al. 2017), supporting a potential contribution of CNS molecular pathways to muscular strength.

We also detected age‐related changes in excitatory and inhibitory synaptic responses evoked by stimulation in layer II/III of the aged motor cortex (Figure 4). Consistent with this, prior evidence shows age‐dependent alterations in layer II/III–layer V synaptic connectivity (Inoue et al. 2023), with aged LVPNs displaying increased dendritic spine density alongside reduced spine survival and increased turnover (Davidson et al. 2020), suggesting reorganization of excitatory inputs onto these neurons with age. We found that EPSC PPR was increased in aged LVPNs, indicating synaptic facilitation and reduced initial release probability at excitatory inputs. While this might appear to reduce excitatory drive, low release probability synapses are better suited to sustain transmission during high‐frequency activity (Gross et al. 2000), as vesicle pools are less likely to deplete during bursts compared to high release probability synapses (Dittman et al. 2000). Aged excitatory synapses may therefore be particularly effective at driving LVPN firing during the high‐frequency activity that characterizes voluntary motor output (Gross et al. 2000; Brecht et al. 2004). EPSC maximum amplitude, tau, and halfwidth measured under 0.5 Hz stimulation were unchanged with age. While these measurements provide information about the amplitude and kinetics of the evoked excitatory response, they do not exclude age‐related changes in glutamate receptor composition or NMDA receptor contribution. Future studies measuring AMPA/NMDA ratio and rectification index will be needed to address these possibilities.

Layer II/III‐evoked inhibitory responses were also altered: IPSC PPR was decreased, suggesting synaptic depression and increased initial release probability—indicating that inhibitory synapses may release strongly on the first pulse but deplete rapidly during sustained activation. IPSC maximum amplitude under 0.5 Hz stimulation was also reduced, indicating a smaller evoked inhibitory response under this stimulation condition independent of short‐term plasticity effects. Together, these synaptic results suggest that, within the inputs recruited by layer II/III stimulation, aged LVPNs exhibited altered excitatory and inhibitory synaptic dynamics during repeated activation. Increased EPSC PPR suggests that recruited excitatory inputs may be better positioned to sustain transmission during high‐frequency activity, whereas decreased IPSC PPR suggests that recruited inhibitory inputs may undergo greater short‐term depression during repeated activation. This pattern of altered synaptic dynamics may become particularly relevant during sustained motor output, potentially contributing to and sustaining the intrinsic hyperexcitability of aged LVPNs observed here. However, these measurements do not establish a global shift in synaptic E/I balance, as they reflect only the excitatory and inhibitory inputs recruited by this stimulation protocol. Further, although our pharmacological experiments confirmed that the recorded responses are synaptic (Figure S3), this approach does not allow us to determine whether the activated inputs originate specifically from layer II/III neurons or distinguish contributions from other fibers such as thalamocortical fibers recruited near the stimulation site. Cell‐type‐ or pathway‐specific approaches, such as optogenetic circuit mapping, will be needed to resolve the specific sources of these synaptic changes.

Finally, our findings raise the possibility that LVPN hyperexcitability may contribute to neuromuscular dysfunction in aging rather than represent a mere correlate. PCA of all cortical, neuromuscular, and behavioral variables revealed clear separation between young and aged mice (Figure 5A), indicating that the combined cortical, neuromuscular, and behavioral profile reliably distinguishes the two age groups. Pearson correlations across all 250 pairwise relationships further showed that cortical excitability and synaptic properties strongly correlated with neuromuscular and behavioral function at the level of individual animals. We found that the large majority of significant correlations were negative across measures ranging from CMAP amplitude to whole‐animal motor function, indicating that animals with higher cortical excitability tend to have worse neuromuscular and behavioral outcomes across the combined young and aged sample (Figure 5B–E). Most strikingly, regression and mediation analyses revealed that LVPN firing frequency statistically accounted for 75% of the age‐related effect on neuromuscular function in our original model (Figure 6C); however, this figure reflects a specific directional assumption, and a reverse‐model analysis (Figure 6D) found a smaller proportion mediated (42.2%) when the mediator and outcome were swapped, highlighting that mediation analysis on observational data cannot establish the direction of this relationship.

The emergence of motor cortex LVPN firing frequency as the dominant statistical correlate of neuromuscular dysfunction is consistent with the established role of LVPNs as a heterogeneous population of deep‐layer projection neurons that contribute to cortical motor output. These neurons fire at high frequencies during voluntary movement (Gross et al. 2000) and action potential trains at 50 Hz in single layer V neurons are sufficient to drive movement (Brecht et al. 2004), suggesting that appropriate scaling of cortical output may be required for coordinated motor function. Consistent with this, OAs exhibit deficits in motor coordination and voluntary muscle activation that have been linked to altered cortical motor output, suggesting that dysregulated cortical drive may contribute to the motor deficits observed in aging (Bhandari et al. 2016). However, whether elevated output from hyperexcitable LVPNs contributes to impaired motor function or represents a compensatory response remains unclear, though recent evidence that chemogenetically inducing motor cortex LVPN hyperexcitability reduces muscular force and coordination in mice (Haidar et al. 2025) raises the possibility that this phenotype may be maladaptive. Notably, LVPN hyperexcitability is not unique to aging—it is a well‐established feature of ALS and is associated with downstream motor deficits (Xie et al. 2023)—suggesting it may be a shared mechanism across distinct contexts of motor dysfunction. Future studies directly manipulating cortical excitability in aged animals—for example by dampening LVPN hyperexcitability—will be necessary to determine whether altering cortical excitability is sufficient to modulate neuromuscular outcomes.

In summary, we identify enhanced cortical motor output and LVPN hyperexcitability as features of the aged motor system that are strongly coupled to neuromuscular dysfunction, with LVPN firing frequency statistically accounting for 75% of the effect of aging on neuromuscular function. This LVPN hyperexcitability was accompanied by altered excitatory and inhibitory synaptic responses evoked by layer II/III stimulation and upregulation of molecular markers of excitability within single neurons of the aged motor cortex. Together, these findings identify the motor cortex as a candidate site for future mechanistic investigation in age‐related weakness, particularly given the growing evidence that hyperexcitability of cortical circuits may be a shared feature of motor dysfunction across neurodegenerative disease contexts.

## Author Contributions

J.A.V., J.M.S., and W.D.A. conceived and designed research. J.A.V., N.R.K., C.D.B., F.B.D., A.R.D., S.N.A., P.J.M., M.W., H.S., B.Y. performed experiments. J.A.V. analyzed data. J.A.V., N.R.K., C.D.B., B.Y., J.M.S., and W.D.A. interpreted results of experiments. J.A.V. prepared figures. J.A.V. drafted manuscript. J.A.V., N.R.K., C.D.B., J.M.S., and W.D.A. edited and revised manuscript. All authors approved final version of manuscript.

## Funding

This project was funded by W. David Arnold: Funding from NIA/NIH (1R01AG067758 and R01AG078129). Joseph M. Santin: R01NS114514 and National Science Foundation‐Award # 2515635.

## Conflicts of Interest

The authors declare no conflicts of interest.

## Supporting information


**Figure S1:** Non‐normalized behavioral measures in young and aged mice.
**Figure S2:** Raw non‐normalized mRNA copy numbers in layer V pyramidal neurons.
**Figure S3:** Pharmacological confirmation of evoked EPSCs and IPSCs, and full‐distribution amplitude histograms.
**Figure S4:** Uncorrected Pearson correlations between cortical and neuromuscular/behavioral variables, young and aged mice analyzed separately.
**Table S1:** Uncorrected Pearson correlations, young mice only.
**Table S2:** Uncorrected Pearson correlations, aged mice only.
**Table S3:** Age × cortical variable interaction analysis, both directions.

## Data Availability

Data are available upon reasonable request to the corresponding author.
