# Supplementary material for: Motor Cortex Hyperexcitability Is Coupled to Neuromuscular Dysfunction in Aged Mice
Source: Aging Cell. 2026 Sep 23;25(10):e70731. doi: 10.1111/acel.70731 (PMC13599356; doi:10.1111/acel.70731)
Supplement: Supplementary file 1 [file ACEL-25-e70731-s001.docx]

**Supporting document 1**

**Figure S1. Non-normalized behavioral measures in young and aged mice.** All-limb grip strength (non-normalized): 219.4 ± 22.73 g (mean ± SD) in young mice vs. 169.2 ± 29.20 g in aged mice; p<0.0001; n=16/group. Weighted-cart pull (non-normalized): 373.3 ± 48.28 g in young mice vs. 284.2 ± 47.02 g in aged mice; p<0.0001; n=16/group.

**Figure S2. Raw non-normalized mRNA copy numbers in layer V pyramidal neurons.** Single-cell transcript analysis was performed in a separate cohort of N = 4 mice per group, with n = 20 LVPNs analyzed per group. (A) 18S (*Rn18s*): 7992 ± 5595 copies/µL in young mice vs. 7118 ± 4532 copies/µL in aged mice; p=0.5902; n=20/group. (B) Nav1.1 (*Scn1a*): 134.6 ± 74.80 copies/µL in young mice vs. 264.2 ± 127.6 copies/µL in aged mice; p=0.0005; n=20/group. Nav1.6 (*Scn8a*): 176.2 ± 231.4 copies/µL in young mice vs. 255.6 ± 142.5 copies/µL in aged mice; p=0.2002; n=20/group. Cav1.3 (*Cacna1d*): 9.784 ± 7.011 copies/µL in young mice vs. 8.323 ± 5.450 copies/µL in aged mice; p=0.4665; n=20/group. 5HT2Cr (*Htr2c*): 12.91 ± 14.41 copies/µL in young mice vs. 136.6 ± 68.45 copies/µL in aged mice; p<0.0001; n=20/group.

**Figure S3. Pharmacological confirmation of evoked EPSCs and IPSCs, and full-distribution amplitude histograms.** (A) Representative traces showing the evoked EPSC (holding potential -70 mV) is abolished by the AMPA receptor antagonist DNQX (20 µM; N= 1, n=3 cells from 3-month old animal) and the evoked IPSC (holding potential 0 mV + TEA) is abolished by the GABA-A receptor antagonist bicuculline (50 µM; N= 1, n=3 cells from 3-month old animal). (B) Full distribution of EPSC and IPSC amplitudes evoked during the 0.5 Hz stimulation protocol, across all recordings, young (gray) and aged (pink). EPSC: median 126.0 pA (mean 169.9 pA, n=77) in young mice vs. median 141.2 pA (mean 188.0 pA, n=83) in aged mice. IPSC: median 422.5 pA (mean 450.5 pA, n=77) in young mice vs. median 260.0 pA (mean 283.3 pA, n=83) in aged mice. Dashed lines indicate the median for each group.

**Figure S4. Uncorrected Pearson correlations between cortical and neuromuscular/behavioral variables, young and aged mice analyzed separately.** (A) Uncorrected Pearson correlations in young mice only (n=16), shown for cortical variables (x-axis) against neuromuscular and behavioral variables (y-axis). Only pairs with uncorrected p<0.05 are shown; r-values displayed. Full r and p values for all pairs are provided in Table S1. (B) Uncorrected Pearson correlations in aged mice only (n=16), same format as (A). Full r and p values for all pairs are provided in Table S2. These correlations are uncorrected for multiple comparisons and are provided for transparency only; they are not used to support claims of statistical significance (see main text and interaction analysis, Table S3).

**Table S1. Uncorrected Pearson correlations, young mice only.** Cortical and neuromuscular/behavioral variables, Pearson r and p-values for all 250 cortical-neuromuscular/behavioral variable pairs, calculated within young mice only (n=16). Values are uncorrected for multiple comparisons.

| **Age** | **cortical** | **Neuromuscular/behavioral** | **r_value** | **p_value** |
| --- | --- | --- | --- | --- |
| **Young** | RIN | CMAP | -0.178 | 0.50899 |
| **Young** | RMP | CMAP | -0.024 | 0.92827 |
| **Young** | Sag Ratio | CMAP | 0.04 | 0.88297 |
| **Young** | AP amplitude | CMAP | -0.244 | 0.36329 |
| **Young** | AP threshold | CMAP | -0.215 | 0.42376 |
| **Young** | AP halfwidth | CMAP | -0.297 | 0.26412 |
| **Young** | AHP | CMAP | -0.031 | 0.90797 |
| **Young** | AP-RMP | CMAP | -0.214 | 0.42531 |
| **Young** | Frequency at 50 pa | CMAP | -0.206 | 0.44482 |
| **Young** | Frequency at 100 pa | CMAP | -0.094 | 0.72904 |
| **Young** | Frequency at 150 pa | CMAP | -0.239 | 0.37245 |
| **Young** | Frequency at 200 pa | CMAP | -0.136 | 0.61545 |
| **Young** | Frequency at 250 pa | CMAP | 0.045 | 0.86802 |
| **Young** | Frequency at 300 pa | CMAP | 0.1 | 0.71198 |
| **Young** | Capacitance | CMAP | 0.051 | 0.85106 |
| **Young** | TAU | CMAP | -0.11 | 0.6852 |
| **Young** | EPSC Amplitude | CMAP | -0.01 | 0.97114 |
| **Young** | EPSC Halfwidth | CMAP | -0.478 | 0.06123 |
| **Young** | EPSC TAU | CMAP | -0.4 | 0.1247 |
| **Young** | EPSC PPR | CMAP | -0.36 | 0.17117 |
| **Young** | IPSC Amplitude | CMAP | 0.517 | 0.04017 |
| **Young** | IPSC Halfwidth | CMAP | -0.099 | 0.71399 |
| **Young** | IPSC TAU | CMAP | 0.066 | 0.80763 |
| **Young** | IPSC PPR | CMAP | 0.03 | 0.91363 |
| **Young** | Cranial MEP | CMAP | -0.269 | 0.31318 |
| **Young** | RIN | SMUP | 0.301 | 0.25755 |
| **Young** | RMP | SMUP | 0.135 | 0.61733 |
| **Young** | Sag Ratio | SMUP | 0.031 | 0.90927 |
| **Young** | AP amplitude | SMUP | -0.093 | 0.7314 |
| **Young** | AP threshold | SMUP | 0.31 | 0.24301 |
| **Young** | AP halfwidth | SMUP | 0.153 | 0.57163 |
| **Young** | AHP | SMUP | 0.028 | 0.91899 |
| **Young** | AP-RMP | SMUP | -0.122 | 0.65274 |
| **Young** | Frequency at 50 pa | SMUP | 0.673 | 0.00426 |
| **Young** | Frequency at 100 pa | SMUP | 0.237 | 0.37591 |
| **Young** | Frequency at 150 pa | SMUP | 0.296 | 0.26516 |
| **Young** | Frequency at 200 pa | SMUP | 0.268 | 0.31608 |
| **Young** | Frequency at 250 pa | SMUP | 0.352 | 0.18182 |
| **Young** | Frequency at 300 pa | SMUP | 0.243 | 0.36435 |
| **Young** | Capacitance | SMUP | -0.42 | 0.10561 |
| **Young** | TAU | SMUP | 0.084 | 0.75753 |
| **Young** | EPSC Amplitude | SMUP | 0.054 | 0.84117 |
| **Young** | EPSC Halfwidth | SMUP | 0.239 | 0.37229 |
| **Young** | EPSC TAU | SMUP | 0.155 | 0.56535 |
| **Young** | EPSC PPR | SMUP | -0.06 | 0.82562 |
| **Young** | IPSC Amplitude | SMUP | -0.204 | 0.44753 |
| **Young** | IPSC Halfwidth | SMUP | 0.049 | 0.85785 |
| **Young** | IPSC TAU | SMUP | -0.08 | 0.76847 |
| **Young** | IPSC PPR | SMUP | -0.173 | 0.52285 |
| **Young** | Cranial MEP | SMUP | -0.139 | 0.60743 |
| **Young** | RIN | MUNE | -0.351 | 0.18264 |
| **Young** | RMP | MUNE | 0.104 | 0.70238 |
| **Young** | Sag Ratio | MUNE | 0.35 | 0.18326 |
| **Young** | AP amplitude | MUNE | 0.054 | 0.84263 |
| **Young** | AP threshold | MUNE | -0.143 | 0.59663 |
| **Young** | AP halfwidth | MUNE | -0.001 | 0.99824 |
| **Young** | AHP | MUNE | -0.08 | 0.7678 |
| **Young** | AP-RMP | MUNE | -0.081 | 0.76478 |
| **Young** | Frequency at 50 pa | MUNE | -0.722 | 0.00159 |
| **Young** | Frequency at 100 pa | MUNE | -0.319 | 0.22905 |
| **Young** | Frequency at 150 pa | MUNE | -0.378 | 0.14841 |
| **Young** | Frequency at 200 pa | MUNE | -0.272 | 0.30729 |
| **Young** | Frequency at 250 pa | MUNE | -0.29 | 0.27552 |
| **Young** | Frequency at 300 pa | MUNE | -0.112 | 0.67841 |
| **Young** | Capacitance | MUNE | 0.587 | 0.01684 |
| **Young** | TAU | MUNE | 0.076 | 0.77958 |
| **Young** | EPSC Amplitude | MUNE | 0.268 | 0.31641 |
| **Young** | EPSC Halfwidth | MUNE | -0.206 | 0.44403 |
| **Young** | EPSC TAU | MUNE | 0.156 | 0.56359 |
| **Young** | EPSC PPR | MUNE | -0.106 | 0.69686 |
| **Young** | IPSC Amplitude | MUNE | 0.617 | 0.01098 |
| **Young** | IPSC Halfwidth | MUNE | 0.1 | 0.71235 |
| **Young** | IPSC TAU | MUNE | -0.18 | 0.50566 |
| **Young** | IPSC PPR | MUNE | 0.144 | 0.59486 |
| **Young** | Cranial MEP | MUNE | 0.34 | 0.19741 |
| **Young** | RIN | RNS | -0.26 | 0.33039 |
| **Young** | RMP | RNS | -0.268 | 0.31546 |
| **Young** | Sag Ratio | RNS | -0.326 | 0.2183 |
| **Young** | AP amplitude | RNS | -0.046 | 0.86609 |
| **Young** | AP threshold | RNS | -0.31 | 0.24248 |
| **Young** | AP halfwidth | RNS | -0.244 | 0.36284 |
| **Young** | AHP | RNS | -0.115 | 0.67051 |
| **Young** | AP-RMP | RNS | -0.29 | 0.27674 |
| **Young** | Frequency at 50 pa | RNS | -0.197 | 0.46507 |
| **Young** | Frequency at 100 pa | RNS | -0.142 | 0.60047 |
| **Young** | Frequency at 150 pa | RNS | -0.247 | 0.35558 |
| **Young** | Frequency at 200 pa | RNS | -0.129 | 0.63363 |
| **Young** | Frequency at 250 pa | RNS | 0.104 | 0.70232 |
| **Young** | Frequency at 300 pa | RNS | 0.104 | 0.70117 |
| **Young** | Capacitance | RNS | -0.015 | 0.95464 |
| **Young** | TAU | RNS | -0.177 | 0.51135 |
| **Young** | EPSC Amplitude | RNS | -0.284 | 0.28576 |
| **Young** | EPSC Halfwidth | RNS | -0.457 | 0.07504 |
| **Young** | EPSC TAU | RNS | -0.697 | 0.0027 |
| **Young** | EPSC PPR | RNS | -0.285 | 0.28387 |
| **Young** | IPSC Amplitude | RNS | -0.043 | 0.87387 |
| **Young** | IPSC Halfwidth | RNS | -0.293 | 0.26992 |
| **Young** | IPSC TAU | RNS | -0.121 | 0.65615 |
| **Young** | IPSC PPR | RNS | 0.229 | 0.39368 |
| **Young** | Cranial MEP | RNS | 0.047 | 0.86286 |
| **Young** | RIN | Cervical MEP | -0.163 | 0.54561 |
| **Young** | RMP | Cervical MEP | -0.408 | 0.11673 |
| **Young** | Sag Ratio | Cervical MEP | -0.379 | 0.14794 |
| **Young** | AP amplitude | Cervical MEP | 0.006 | 0.98198 |
| **Young** | AP threshold | Cervical MEP | -0.492 | 0.05305 |
| **Young** | AP halfwidth | Cervical MEP | -0.263 | 0.32572 |
| **Young** | AHP | Cervical MEP | 0.138 | 0.61109 |
| **Young** | AP-RMP | Cervical MEP | 0.177 | 0.51243 |
| **Young** | Frequency at 50 pa | Cervical MEP | -0.369 | 0.16011 |
| **Young** | Frequency at 100 pa | Cervical MEP | -0.162 | 0.54995 |
| **Young** | Frequency at 150 pa | Cervical MEP | -0.301 | 0.25677 |
| **Young** | Frequency at 200 pa | Cervical MEP | -0.311 | 0.24164 |
| **Young** | Frequency at 250 pa | Cervical MEP | -0.269 | 0.31448 |
| **Young** | Frequency at 300 pa | Cervical MEP | -0.192 | 0.47733 |
| **Young** | Capacitance | Cervical MEP | -0.202 | 0.4526 |
| **Young** | TAU | Cervical MEP | -0.371 | 0.15727 |
| **Young** | EPSC Amplitude | Cervical MEP | -0.229 | 0.39449 |
| **Young** | EPSC Halfwidth | Cervical MEP | -0.479 | 0.06054 |
| **Young** | EPSC TAU | Cervical MEP | -0.593 | 0.01542 |
| **Young** | EPSC PPR | Cervical MEP | -0.203 | 0.44992 |
| **Young** | IPSC Amplitude | Cervical MEP | -0.145 | 0.593 |
| **Young** | IPSC Halfwidth | Cervical MEP | -0.047 | 0.86155 |
| **Young** | IPSC TAU | Cervical MEP | 0.253 | 0.34469 |
| **Young** | IPSC PPR | Cervical MEP | 0.089 | 0.74191 |
| **Young** | Cranial MEP | Cervical MEP | -0.039 | 0.88525 |
| **Young** | RIN | Twitch | -0.223 | 0.40542 |
| **Young** | RMP | Twitch | 0.422 | 0.1035 |
| **Young** | Sag Ratio | Twitch | 0.486 | 0.05614 |
| **Young** | AP amplitude | Twitch | -0.364 | 0.16544 |
| **Young** | AP threshold | Twitch | 0.324 | 0.22029 |
| **Young** | AP halfwidth | Twitch | 0.107 | 0.69422 |
| **Young** | AHP | Twitch | -0.504 | 0.04632 |
| **Young** | AP-RMP | Twitch | -0.025 | 0.92631 |
| **Young** | Frequency at 50 pa | Twitch | -0.276 | 0.29992 |
| **Young** | Frequency at 100 pa | Twitch | -0.265 | 0.32081 |
| **Young** | Frequency at 150 pa | Twitch | -0.265 | 0.32149 |
| **Young** | Frequency at 200 pa | Twitch | -0.143 | 0.59704 |
| **Young** | Frequency at 250 pa | Twitch | -0.139 | 0.60775 |
| **Young** | Frequency at 300 pa | Twitch | -0.064 | 0.81414 |
| **Young** | Capacitance | Twitch | 0.248 | 0.35391 |
| **Young** | TAU | Twitch | 0.55 | 0.02738 |
| **Young** | EPSC Amplitude | Twitch | 0.133 | 0.62306 |
| **Young** | EPSC Halfwidth | Twitch | -0.031 | 0.90963 |
| **Young** | EPSC TAU | Twitch | 0.249 | 0.35275 |
| **Young** | EPSC PPR | Twitch | 0.467 | 0.06798 |
| **Young** | IPSC Amplitude | Twitch | 0.467 | 0.06805 |
| **Young** | IPSC Halfwidth | Twitch | -0.116 | 0.66907 |
| **Young** | IPSC TAU | Twitch | -0.491 | 0.05331 |
| **Young** | IPSC PPR | Twitch | 0.062 | 0.81939 |
| **Young** | Cranial MEP | Twitch | 0.299 | 0.26004 |
| **Young** | RIN | Tetanic | 0.015 | 0.95459 |
| **Young** | RMP | Tetanic | -0.404 | 0.12043 |
| **Young** | Sag Ratio | Tetanic | -0.148 | 0.58514 |
| **Young** | AP amplitude | Tetanic | -0.068 | 0.80379 |
| **Young** | AP threshold | Tetanic | -0.204 | 0.44749 |
| **Young** | AP halfwidth | Tetanic | 0.003 | 0.99247 |
| **Young** | AHP | Tetanic | 0.07 | 0.79612 |
| **Young** | AP-RMP | Tetanic | 0.091 | 0.73691 |
| **Young** | Frequency at 50 pa | Tetanic | 0.161 | 0.55105 |
| **Young** | Frequency at 100 pa | Tetanic | 0.079 | 0.76983 |
| **Young** | Frequency at 150 pa | Tetanic | -0.024 | 0.92851 |
| **Young** | Frequency at 200 pa | Tetanic | 0.031 | 0.90873 |
| **Young** | Frequency at 250 pa | Tetanic | 0.105 | 0.69971 |
| **Young** | Frequency at 300 pa | Tetanic | 0.065 | 0.80984 |
| **Young** | Capacitance | Tetanic | -0.258 | 0.33374 |
| **Young** | TAU | Tetanic | 0.17 | 0.52974 |
| **Young** | EPSC Amplitude | Tetanic | -0.302 | 0.25552 |
| **Young** | EPSC Halfwidth | Tetanic | -0.344 | 0.19254 |
| **Young** | EPSC TAU | Tetanic | -0.329 | 0.21337 |
| **Young** | EPSC PPR | Tetanic | -0.176 | 0.51326 |
| **Young** | IPSC Amplitude | Tetanic | -0.071 | 0.79524 |
| **Young** | IPSC Halfwidth | Tetanic | -0.321 | 0.22502 |
| **Young** | IPSC TAU | Tetanic | -0.138 | 0.60944 |
| **Young** | IPSC PPR | Tetanic | -0.13 | 0.63193 |
| **Young** | Cranial MEP | Tetanic | -0.516 | 0.04071 |
| **Young** | RIN | ROTAROD | -0.097 | 0.72003 |
| **Young** | RMP | ROTAROD | 0.2 | 0.45875 |
| **Young** | Sag Ratio | ROTAROD | -0.121 | 0.65538 |
| **Young** | AP amplitude | ROTAROD | -0.4 | 0.1243 |
| **Young** | AP threshold | ROTAROD | 0.204 | 0.44842 |
| **Young** | AP halfwidth | ROTAROD | -0.034 | 0.89946 |
| **Young** | AHP | ROTAROD | -0.25 | 0.35068 |
| **Young** | AP-RMP | ROTAROD | -0.008 | 0.97508 |
| **Young** | Frequency at 50 pa | ROTAROD | -0.192 | 0.47615 |
| **Young** | Frequency at 100 pa | ROTAROD | 0.228 | 0.39565 |
| **Young** | Frequency at 150 pa | ROTAROD | -0.081 | 0.76576 |
| **Young** | Frequency at 200 pa | ROTAROD | -0.049 | 0.85725 |
| **Young** | Frequency at 250 pa | ROTAROD | 0.128 | 0.63778 |
| **Young** | Frequency at 300 pa | ROTAROD | -0.042 | 0.87719 |
| **Young** | Capacitance | ROTAROD | -0.17 | 0.52944 |
| **Young** | TAU | ROTAROD | 0.325 | 0.21875 |
| **Young** | EPSC Amplitude | ROTAROD | 0.324 | 0.22038 |
| **Young** | EPSC Halfwidth | ROTAROD | -0.286 | 0.28294 |
| **Young** | EPSC TAU | ROTAROD | -0.307 | 0.24784 |
| **Young** | EPSC PPR | ROTAROD | 0.127 | 0.63846 |
| **Young** | IPSC Amplitude | ROTAROD | -0.192 | 0.47648 |
| **Young** | IPSC Halfwidth | ROTAROD | 0.069 | 0.79886 |
| **Young** | IPSC TAU | ROTAROD | -0.254 | 0.34201 |
| **Young** | IPSC PPR | ROTAROD | 0.267 | 0.31713 |
| **Young** | Cranial MEP | ROTAROD | 0.087 | 0.74903 |
| **Young** | RIN | Grip Strength | 0.056 | 0.83741 |
| **Young** | RMP | Grip Strength | 0.042 | 0.87636 |
| **Young** | Sag Ratio | Grip Strength | 0.164 | 0.54339 |
| **Young** | AP amplitude | Grip Strength | -0.105 | 0.69967 |
| **Young** | AP threshold | Grip Strength | -0.102 | 0.7075 |
| **Young** | AP halfwidth | Grip Strength | 0.209 | 0.43785 |
| **Young** | AHP | Grip Strength | 0.077 | 0.77742 |
| **Young** | AP-RMP | Grip Strength | -0.045 | 0.86725 |
| **Young** | Frequency at 50 pa | Grip Strength | -0.241 | 0.36923 |
| **Young** | Frequency at 100 pa | Grip Strength | -0.203 | 0.45072 |
| **Young** | Frequency at 150 pa | Grip Strength | -0.631 | 0.00874 |
| **Young** | Frequency at 200 pa | Grip Strength | -0.662 | 0.00519 |
| **Young** | Frequency at 250 pa | Grip Strength | -0.616 | 0.01105 |
| **Young** | Frequency at 300 pa | Grip Strength | -0.681 | 0.00367 |
| **Young** | Capacitance | Grip Strength | 0.157 | 0.56102 |
| **Young** | TAU | Grip Strength | 0.541 | 0.03047 |
| **Young** | EPSC Amplitude | Grip Strength | -0.187 | 0.4875 |
| **Young** | EPSC Halfwidth | Grip Strength | -0.217 | 0.41842 |
| **Young** | EPSC TAU | Grip Strength | -0.113 | 0.67729 |
| **Young** | EPSC PPR | Grip Strength | 0.312 | 0.2387 |
| **Young** | IPSC Amplitude | Grip Strength | 0.202 | 0.45234 |
| **Young** | IPSC Halfwidth | Grip Strength | 0.087 | 0.74805 |
| **Young** | IPSC TAU | Grip Strength | 0.11 | 0.68506 |
| **Young** | IPSC PPR | Grip Strength | -0.351 | 0.18306 |
| **Young** | Cranial MEP | Grip Strength | -0.026 | 0.92502 |
| **Young** | RIN | Weighted pull | -0.356 | 0.17539 |
| **Young** | RMP | Weighted pull | -0.051 | 0.85129 |
| **Young** | Sag Ratio | Weighted pull | 0.324 | 0.22111 |
| **Young** | AP amplitude | Weighted pull | -0.339 | 0.19965 |
| **Young** | AP threshold | Weighted pull | -0.222 | 0.40836 |
| **Young** | AP halfwidth | Weighted pull | -0.222 | 0.40812 |
| **Young** | AHP | Weighted pull | -0.104 | 0.70286 |
| **Young** | AP-RMP | Weighted pull | 0.389 | 0.13675 |
| **Young** | Frequency at 50 pa | Weighted pull | -0.325 | 0.21994 |
| **Young** | Frequency at 100 pa | Weighted pull | -0.325 | 0.21919 |
| **Young** | Frequency at 150 pa | Weighted pull | -0.387 | 0.13894 |
| **Young** | Frequency at 200 pa | Weighted pull | -0.351 | 0.18257 |
| **Young** | Frequency at 250 pa | Weighted pull | -0.317 | 0.23224 |
| **Young** | Frequency at 300 pa | Weighted pull | -0.216 | 0.421 |
| **Young** | Capacitance | Weighted pull | -0.127 | 0.63894 |
| **Young** | TAU | Weighted pull | 0.279 | 0.2953 |
| **Young** | EPSC Amplitude | Weighted pull | 0.083 | 0.75923 |
| **Young** | EPSC Halfwidth | Weighted pull | -0.474 | 0.06334 |
| **Young** | EPSC TAU | Weighted pull | -0.137 | 0.6134 |
| **Young** | EPSC PPR | Weighted pull | 0.274 | 0.30401 |
| **Young** | IPSC Amplitude | Weighted pull | 0.354 | 0.17858 |
| **Young** | IPSC Halfwidth | Weighted pull | -0.073 | 0.78692 |
| **Young** | IPSC TAU | Weighted pull | -0.217 | 0.41907 |
| **Young** | IPSC PPR | Weighted pull | 0.047 | 0.8622 |
| **Young** | Cranial MEP | Weighted pull | 0.114 | 0.67333 |

**Table S2. Uncorrected Pearson correlations, aged mice only.** Cortical and neuromuscular/behavioral variables, Pearson r and p-values for all 250 cortical-neuromuscular/behavioral variable pairs, calculated within young mice only (n=16). Values are uncorrected for multiple comparisons.

| **Age** | **cortical** | **Neuromuscular/behavioral** | **r_value** | **p_value** |
| --- | --- | --- | --- | --- |
| **Old** | RIN | CMAP | -0.081 | 0.76614 |
| **Old** | RMP | CMAP | -0.21 | 0.43432 |
| **Old** | Sag Ratio | CMAP | 0.284 | 0.2864 |
| **Old** | AP amplitude | CMAP | -0.418 | 0.10697 |
| **Old** | AP threshold | CMAP | 0.123 | 0.65084 |
| **Old** | AP halfwidth | CMAP | -0.025 | 0.92752 |
| **Old** | AHP | CMAP | 0.262 | 0.32748 |
| **Old** | AP-RMP | CMAP | 0.135 | 0.61718 |
| **Old** | Frequency at 50 pa | CMAP | -0.657 | 0.00566 |
| **Old** | Frequency at 100 pa | CMAP | -0.425 | 0.10102 |
| **Old** | Frequency at 150 pa | CMAP | -0.101 | 0.70985 |
| **Old** | Frequency at 200 pa | CMAP | -0.143 | 0.5982 |
| **Old** | Frequency at 250 pa | CMAP | -0.181 | 0.50206 |
| **Old** | Frequency at 300 pa | CMAP | -0.243 | 0.36354 |
| **Old** | Capacitance | CMAP | 0.08 | 0.7684 |
| **Old** | TAU | CMAP | 0.154 | 0.5696 |
| **Old** | EPSC Amplitude | CMAP | 0.003 | 0.99148 |
| **Old** | EPSC Halfwidth | CMAP | 0.41 | 0.11501 |
| **Old** | EPSC TAU | CMAP | -0.028 | 0.91819 |
| **Old** | EPSC PPR | CMAP | -0.699 | 0.00259 |
| **Old** | IPSC Amplitude | CMAP | 0.148 | 0.58506 |
| **Old** | IPSC Halfwidth | CMAP | -0.093 | 0.7323 |
| **Old** | IPSC TAU | CMAP | -0.106 | 0.69648 |
| **Old** | IPSC PPR | CMAP | 0.411 | 0.11361 |
| **Old** | Cranial MEP | CMAP | 0.219 | 0.41558 |
| **Old** | RIN | SMUP | 0.326 | 0.21749 |
| **Old** | RMP | SMUP | 0.002 | 0.9939 |
| **Old** | Sag Ratio | SMUP | 0.007 | 0.97908 |
| **Old** | AP amplitude | SMUP | 0.249 | 0.35227 |
| **Old** | AP threshold | SMUP | -0.462 | 0.07169 |
| **Old** | AP halfwidth | SMUP | -0.121 | 0.65626 |
| **Old** | AHP | SMUP | 0.094 | 0.72818 |
| **Old** | AP-RMP | SMUP | 0.114 | 0.67343 |
| **Old** | Frequency at 50 pa | SMUP | -0.133 | 0.62345 |
| **Old** | Frequency at 100 pa | SMUP | -0.33 | 0.21214 |
| **Old** | Frequency at 150 pa | SMUP | -0.515 | 0.04102 |
| **Old** | Frequency at 200 pa | SMUP | -0.375 | 0.15212 |
| **Old** | Frequency at 250 pa | SMUP | -0.186 | 0.4893 |
| **Old** | Frequency at 300 pa | SMUP | -0.131 | 0.62961 |
| **Old** | Capacitance | SMUP | -0.002 | 0.99425 |
| **Old** | TAU | SMUP | -0.009 | 0.97339 |
| **Old** | EPSC Amplitude | SMUP | -0.254 | 0.34316 |
| **Old** | EPSC Halfwidth | SMUP | 0.017 | 0.94997 |
| **Old** | EPSC TAU | SMUP | 0.102 | 0.70701 |
| **Old** | EPSC PPR | SMUP | -0.058 | 0.83086 |
| **Old** | IPSC Amplitude | SMUP | 0.33 | 0.21242 |
| **Old** | IPSC Halfwidth | SMUP | 0.262 | 0.32767 |
| **Old** | IPSC TAU | SMUP | 0.35 | 0.18372 |
| **Old** | IPSC PPR | SMUP | -0.021 | 0.93962 |
| **Old** | Cranial MEP | SMUP | -0.096 | 0.72324 |
| **Old** | RIN | MUNE | -0.505 | 0.04616 |
| **Old** | RMP | MUNE | -0.111 | 0.68353 |
| **Old** | Sag Ratio | MUNE | -0.041 | 0.8797 |
| **Old** | AP amplitude | MUNE | -0.432 | 0.09433 |
| **Old** | AP threshold | MUNE | 0.468 | 0.06722 |
| **Old** | AP halfwidth | MUNE | 0.216 | 0.42075 |
| **Old** | AHP | MUNE | 0.061 | 0.82361 |
| **Old** | AP-RMP | MUNE | 0.117 | 0.66631 |
| **Old** | Frequency at 50 pa | MUNE | -0.474 | 0.06353 |
| **Old** | Frequency at 100 pa | MUNE | -0.208 | 0.43988 |
| **Old** | Frequency at 150 pa | MUNE | 0.14 | 0.60478 |
| **Old** | Frequency at 200 pa | MUNE | -0.018 | 0.94614 |
| **Old** | Frequency at 250 pa | MUNE | -0.248 | 0.35426 |
| **Old** | Frequency at 300 pa | MUNE | -0.338 | 0.20105 |
| **Old** | Capacitance | MUNE | 0.205 | 0.44722 |
| **Old** | TAU | MUNE | 0.238 | 0.37466 |
| **Old** | EPSC Amplitude | MUNE | 0.13 | 0.63105 |
| **Old** | EPSC Halfwidth | MUNE | 0.266 | 0.31844 |
| **Old** | EPSC TAU | MUNE | 0.063 | 0.81564 |
| **Old** | EPSC PPR | MUNE | -0.536 | 0.03219 |
| **Old** | IPSC Amplitude | MUNE | -0.045 | 0.86917 |
| **Old** | IPSC Halfwidth | MUNE | 0.05 | 0.85474 |
| **Old** | IPSC TAU | MUNE | -0.3 | 0.25847 |
| **Old** | IPSC PPR | MUNE | 0.254 | 0.34221 |
| **Old** | Cranial MEP | MUNE | 0.194 | 0.47209 |
| **Old** | RIN | RNS | 0.328 | 0.21560 |
| **Old** | RMP | RNS | -0.004 | 0.98744 |
| **Old** | Sag Ratio | RNS | 0.201 | 0.45546 |
| **Old** | AP amplitude | RNS | 0.545 | 0.02892 |
| **Old** | AP threshold | RNS | -0.442 | 0.08617 |
| **Old** | AP halfwidth | RNS | -0.084 | 0.7582 |
| **Old** | AHP | RNS | -0.319 | 0.22903 |
| **Old** | AP-RMP | RNS | -0.405 | 0.11975 |
| **Old** | Frequency at 50 pa | RNS | 0.182 | 0.4992 |
| **Old** | Frequency at 100 pa | RNS | -0.185 | 0.49281 |
| **Old** | Frequency at 150 pa | RNS | -0.073 | 0.78855 |
| **Old** | Frequency at 200 pa | RNS | -0.101 | 0.71112 |
| **Old** | Frequency at 250 pa | RNS | -0.093 | 0.73195 |
| **Old** | Frequency at 300 pa | RNS | -0.102 | 0.70778 |
| **Old** | Capacitance | RNS | 0.036 | 0.89391 |
| **Old** | TAU | RNS | -0.049 | 0.85668 |
| **Old** | EPSC Amplitude | RNS | 0.042 | 0.87816 |
| **Old** | EPSC Halfwidth | RNS | -0.389 | 0.1361 |
| **Old** | EPSC TAU | RNS | -0.265 | 0.32197 |
| **Old** | EPSC PPR | RNS | -0.006 | 0.98286 |
| **Old** | IPSC Amplitude | RNS | 0.167 | 0.53577 |
| **Old** | IPSC Halfwidth | RNS | 0.186 | 0.49033 |
| **Old** | IPSC TAU | RNS | -0.061 | 0.82243 |
| **Old** | IPSC PPR | RNS | -0.5 | 0.04873 |
| **Old** | Cranial MEP | RNS | -0.224 | 0.40419 |
| **Old** | RIN | Cervical MEP | -0.435 | 0.09202 |
| **Old** | RMP | Cervical MEP | 0.6 | 0.01394 |
| **Old** | Sag Ratio | Cervical MEP | -0.384 | 0.1422 |
| **Old** | AP amplitude | Cervical MEP | 0.182 | 0.501 |
| **Old** | AP threshold | Cervical MEP | 0.484 | 0.05777 |
| **Old** | AP halfwidth | Cervical MEP | -0.082 | 0.7624 |
| **Old** | AHP | Cervical MEP | -0.128 | 0.63622 |
| **Old** | AP-RMP | Cervical MEP | -0.148 | 0.58359 |
| **Old** | Frequency at 50 pa | Cervical MEP | -0.322 | 0.22328 |
| **Old** | Frequency at 100 pa | Cervical MEP | -0.128 | 0.63596 |
| **Old** | Frequency at 150 pa | Cervical MEP | 0.381 | 0.14566 |
| **Old** | Frequency at 200 pa | Cervical MEP | 0.153 | 0.57159 |
| **Old** | Frequency at 250 pa | Cervical MEP | 0.014 | 0.95916 |
| **Old** | Frequency at 300 pa | Cervical MEP | 0.14 | 0.60572 |
| **Old** | Capacitance | Cervical MEP | 0.009 | 0.9735 |
| **Old** | TAU | Cervical MEP | 0.021 | 0.9399 |
| **Old** | EPSC Amplitude | Cervical MEP | -0.174 | 0.51863 |
| **Old** | EPSC Halfwidth | Cervical MEP | -0.163 | 0.54575 |
| **Old** | EPSC TAU | Cervical MEP | -0.021 | 0.93977 |
| **Old** | EPSC PPR | Cervical MEP | -0.149 | 0.58278 |
| **Old** | IPSC Amplitude | Cervical MEP | -0.477 | 0.06166 |
| **Old** | IPSC Halfwidth | Cervical MEP | 0.315 | 0.23448 |
| **Old** | IPSC TAU | Cervical MEP | -0.303 | 0.2545 |
| **Old** | IPSC PPR | Cervical MEP | 0.407 | 0.11802 |
| **Old** | Cranial MEP | Cervical MEP | 0.15 | 0.57797 |
| **Old** | RIN | Twitch | 0.018 | 0.94838 |
| **Old** | RMP | Twitch | 0.436 | 0.09103 |
| **Old** | Sag Ratio | Twitch | -0.12 | 0.65838 |
| **Old** | AP amplitude | Twitch | 0.685 | 0.00343 |
| **Old** | AP threshold | Twitch | 0.029 | 0.91618 |
| **Old** | AP halfwidth | Twitch | -0.19 | 0.48064 |
| **Old** | AHP | Twitch | -0.196 | 0.46627 |
| **Old** | AP-RMP | Twitch | -0.386 | 0.1403 |
| **Old** | Frequency at 50 pa | Twitch | 0.054 | 0.84204 |
| **Old** | Frequency at 100 pa | Twitch | 0.018 | 0.94592 |
| **Old** | Frequency at 150 pa | Twitch | 0.29 | 0.27587 |
| **Old** | Frequency at 200 pa | Twitch | 0.272 | 0.30877 |
| **Old** | Frequency at 250 pa | Twitch | 0.382 | 0.14461 |
| **Old** | Frequency at 300 pa | Twitch | 0.336 | 0.20364 |
| **Old** | Capacitance | Twitch | 0.07 | 0.79607 |
| **Old** | TAU | Twitch | 0.005 | 0.98405 |
| **Old** | EPSC Amplitude | Twitch | 0.019 | 0.9457 |
| **Old** | EPSC Halfwidth | Twitch | -0.012 | 0.96594 |
| **Old** | EPSC TAU | Twitch | 0.144 | 0.59372 |
| **Old** | EPSC PPR | Twitch | 0.105 | 0.69914 |
| **Old** | IPSC Amplitude | Twitch | -0.313 | 0.23861 |
| **Old** | IPSC Halfwidth | Twitch | 0.244 | 0.3624 |
| **Old** | IPSC TAU | Twitch | -0.323 | 0.22292 |
| **Old** | IPSC PPR | Twitch | 0.101 | 0.70998 |
| **Old** | Cranial MEP | Twitch | 0.022 | 0.93432 |
| **Old** | RIN | Tetanic | 0.223 | 0.40751 |
| **Old** | RMP | Tetanic | 0.093 | 0.73081 |
| **Old** | Sag Ratio | Tetanic | 0.281 | 0.29092 |
| **Old** | AP amplitude | Tetanic | 0.386 | 0.14024 |
| **Old** | AP threshold | Tetanic | 0.031 | 0.91032 |
| **Old** | AP halfwidth | Tetanic | -0.443 | 0.08536 |
| **Old** | AHP | Tetanic | -0.029 | 0.91397 |
| **Old** | AP-RMP | Tetanic | -0.145 | 0.59114 |
| **Old** | Frequency at 50 pa | Tetanic | -0.188 | 0.48504 |
| **Old** | Frequency at 100 pa | Tetanic | -0.328 | 0.21499 |
| **Old** | Frequency at 150 pa | Tetanic | -0.129 | 0.63416 |
| **Old** | Frequency at 200 pa | Tetanic | 0.027 | 0.91999 |
| **Old** | Frequency at 250 pa | Tetanic | 0.198 | 0.46258 |
| **Old** | Frequency at 300 pa | Tetanic | -0.016 | 0.95364 |
| **Old** | Capacitance | Tetanic | 0.05 | 0.85287 |
| **Old** | TAU | Tetanic | 0.182 | 0.50014 |
| **Old** | EPSC Amplitude | Tetanic | 0.198 | 0.46299 |
| **Old** | EPSC Halfwidth | Tetanic | 0.25 | 0.35123 |
| **Old** | EPSC TAU | Tetanic | 0.112 | 0.68089 |
| **Old** | EPSC PPR | Tetanic | -0.572 | 0.02054 |
| **Old** | IPSC Amplitude | Tetanic | 0.042 | 0.87685 |
| **Old** | IPSC Halfwidth | Tetanic | -0.033 | 0.9042 |
| **Old** | IPSC TAU | Tetanic | -0.374 | 0.15355 |
| **Old** | IPSC PPR | Tetanic | 0.011 | 0.96704 |
| **Old** | Cranial MEP | Tetanic | 0.406 | 0.11864 |
| **Old** | RIN | ROTAROD | -0.065 | 0.81202 |
| **Old** | RMP | ROTAROD | -0.46 | 0.07271 |
| **Old** | Sag Ratio | ROTAROD | 0.212 | 0.43001 |
| **Old** | AP amplitude | ROTAROD | -0.365 | 0.16419 |
| **Old** | AP threshold | ROTAROD | 0.089 | 0.74199 |
| **Old** | AP halfwidth | ROTAROD | 0.165 | 0.54156 |
| **Old** | AHP | ROTAROD | -0.138 | 0.61115 |
| **Old** | AP-RMP | ROTAROD | 0.537 | 0.03184 |
| **Old** | Frequency at 50 pa | ROTAROD | -0.289 | 0.27724 |
| **Old** | Frequency at 100 pa | ROTAROD | -0.497 | 0.0502 |
| **Old** | Frequency at 150 pa | ROTAROD | -0.516 | 0.04058 |
| **Old** | Frequency at 200 pa | ROTAROD | -0.548 | 0.02793 |
| **Old** | Frequency at 250 pa | ROTAROD | -0.734 | 0.0012 |
| **Old** | Frequency at 300 pa | ROTAROD | -0.581 | 0.01821 |
| **Old** | Capacitance | ROTAROD | 0.256 | 0.33954 |
| **Old** | TAU | ROTAROD | 0.125 | 0.64477 |
| **Old** | EPSC Amplitude | ROTAROD | -0.069 | 0.8006 |
| **Old** | EPSC Halfwidth | ROTAROD | -0.231 | 0.389 |
| **Old** | EPSC TAU | ROTAROD | -0.317 | 0.2318 |
| **Old** | EPSC PPR | ROTAROD | -0.337 | 0.20177 |
| **Old** | IPSC Amplitude | ROTAROD | 0.432 | 0.09461 |
| **Old** | IPSC Halfwidth | ROTAROD | 0.177 | 0.51259 |
| **Old** | IPSC TAU | ROTAROD | 0.255 | 0.34044 |
| **Old** | IPSC PPR | ROTAROD | -0.071 | 0.79325 |
| **Old** | Cranial MEP | ROTAROD | -0.275 | 0.3026 |
| **Old** | RIN | Grip Strength | 0.504 | 0.04675 |
| **Old** | RMP | Grip Strength | -0.221 | 0.41136 |
| **Old** | Sag Ratio | Grip Strength | 0.433 | 0.09415 |
| **Old** | AP amplitude | Grip Strength | -0.187 | 0.48819 |
| **Old** | AP threshold | Grip Strength | -0.307 | 0.2478 |
| **Old** | AP halfwidth | Grip Strength | -0.525 | 0.03658 |
| **Old** | AHP | Grip Strength | 0.061 | 0.82318 |
| **Old** | AP-RMP | Grip Strength | 0.169 | 0.53208 |
| **Old** | Frequency at 50 pa | Grip Strength | -0.269 | 0.31414 |
| **Old** | Frequency at 100 pa | Grip Strength | -0.492 | 0.05314 |
| **Old** | Frequency at 150 pa | Grip Strength | -0.457 | 0.07547 |
| **Old** | Frequency at 200 pa | Grip Strength | -0.433 | 0.09397 |
| **Old** | Frequency at 250 pa | Grip Strength | -0.305 | 0.25044 |
| **Old** | Frequency at 300 pa | Grip Strength | -0.297 | 0.26383 |
| **Old** | Capacitance | Grip Strength | -0.015 | 0.95563 |
| **Old** | TAU | Grip Strength | 0.059 | 0.82874 |
| **Old** | EPSC Amplitude | Grip Strength | 0.159 | 0.5572 |
| **Old** | EPSC Halfwidth | Grip Strength | 0.029 | 0.91504 |
| **Old** | EPSC TAU | Grip Strength | -0.249 | 0.35268 |
| **Old** | EPSC PPR | Grip Strength | -0.63 | 0.00887 |
| **Old** | IPSC Amplitude | Grip Strength | 0.375 | 0.1523 |
| **Old** | IPSC Halfwidth | Grip Strength | -0.255 | 0.34081 |
| **Old** | IPSC TAU | Grip Strength | 0.171 | 0.52652 |
| **Old** | IPSC PPR | Grip Strength | -0.38 | 0.14708 |
| **Old** | Cranial MEP | Grip Strength | 0.183 | 0.49704 |
| **Old** | RIN | Weighted pull | -0.483 | 0.05834 |
| **Old** | RMP | Weighted pull | 0.166 | 0.53981 |
| **Old** | Sag Ratio | Weighted pull | -0.191 | 0.47913 |
| **Old** | AP amplitude | Weighted pull | -0.133 | 0.62406 |
| **Old** | AP threshold | Weighted pull | 0.475 | 0.06284 |
| **Old** | AP halfwidth | Weighted pull | -0.037 | 0.89304 |
| **Old** | AHP | Weighted pull | 0.291 | 0.2734 |
| **Old** | AP-RMP | Weighted pull | 0.025 | 0.92571 |
| **Old** | Frequency at 50 pa | Weighted pull | -0.201 | 0.45425 |
| **Old** | Frequency at 100 pa | Weighted pull | -0.053 | 0.8444 |
| **Old** | Frequency at 150 pa | Weighted pull | 0.103 | 0.70476 |
| **Old** | Frequency at 200 pa | Weighted pull | -0.05 | 0.85482 |
| **Old** | Frequency at 250 pa | Weighted pull | -0.133 | 0.62376 |
| **Old** | Frequency at 300 pa | Weighted pull | -0.272 | 0.30865 |
| **Old** | Capacitance | Weighted pull | -0.063 | 0.81551 |
| **Old** | TAU | Weighted pull | -0.001 | 0.99738 |
| **Old** | EPSC Amplitude | Weighted pull | 0.391 | 0.13441 |
| **Old** | EPSC Halfwidth | Weighted pull | 0.508 | 0.04447 |
| **Old** | EPSC TAU | Weighted pull | 0.381 | 0.14554 |
| **Old** | EPSC PPR | Weighted pull | -0.425 | 0.10065 |
| **Old** | IPSC Amplitude | Weighted pull | -0.164 | 0.54344 |
| **Old** | IPSC Halfwidth | Weighted pull | 0.11 | 0.6839 |
| **Old** | IPSC TAU | Weighted pull | -0.225 | 0.40158 |
| **Old** | IPSC PPR | Weighted pull | 0.118 | 0.66413 |
| **Old** | Cranial MEP | Weighted pull | 0.488 | 0.05499 |

**Table S3. Age × cortical variable interaction analysis, both directions.** For each cortical-neuromuscular/behavioral variable pair (n=250), a linear model with an interaction term (cortical variable × Age) was fit using the full sample (n=32) to test whether the slope of the relationship between the two variables differs between young and aged mice. Two model directions are reported: neuromuscular/behavioral variable ~ cortical variable × Age, and cortical variable ~ neuromuscular/behavioral variable × Age, to confirm results do not depend on which variable is treated as the outcome. Interaction p-values and FDR-adjusted p-values (Benjamini-Hochberg) are shown for both directions. "Age-dependent" indicates FDR-adjusted p<0.05, meaning the slope differs significantly between young and aged mice for that pair. "Both directions agree" indicates whether the two model directions gave the same conclusion. No pair reached significance in either direction after FDR correction (0 of 250), indicating the slope of these relationships does not differ significantly by age.

| **Cortical variable** | **Neuromuscular/behavioral variable** | **Interaction p-value (Neuromuscular/behavioral ~ cortical × Age)** | **Interaction p-value, FDR-adjusted (Neuromuscular/behavioral ~ cortical × Age)** | **Age-dependent? (Neuromuscular/behavioral ~ cortical × Age)** | **Interaction p-value (cortical ~ Neuromuscular/behavioral × Age)** | **Interaction p-value, FDR-adjusted (cortical ~ Neuromuscular/behavioral × Age)** | **Age-dependent? (cortical ~ Neuromuscular/behavioral × Age)** | **Both directions agree?** |
| --- | --- | --- | --- | --- | --- | --- | --- | --- |
| **RIN** | CMAP | 0.85505 | 0.97695 | FALSE | 0.48629 | 0.93675 | FALSE | TRUE |
| **RMP** | CMAP | 0.40623 | 0.92553 | FALSE | 0.87757 | 0.96417 | FALSE | TRUE |
| **Sag Ratio** | CMAP | 0.66349 | 0.95518 | FALSE | 0.55047 | 0.93675 | FALSE | TRUE |
| **AP amplitude** | CMAP | 0.45343 | 0.92553 | FALSE | 0.88017 | 0.96417 | FALSE | TRUE |
| **AP threshold** | CMAP | 0.43332 | 0.92553 | FALSE | 0.34368 | 0.91524 | FALSE | TRUE |
| **AP halfwidth** | CMAP | 0.58579 | 0.95471 | FALSE | 0.45873 | 0.93675 | FALSE | TRUE |
| **AHP** | CMAP | 0.2942 | 0.89335 | FALSE | 0.61707 | 0.95227 | FALSE | TRUE |
| **AP-RMP** | CMAP | 0.41606 | 0.92553 | FALSE | 0.37249 | 0.91524 | FALSE | TRUE |
| **Frequency at 50 pa** | CMAP | 0.01526 | 0.56639 | FALSE | 0.7726 | 0.95285 | FALSE | TRUE |
| **Frequency at 100 pa** | CMAP | 0.07364 | 0.75452 | FALSE | 0.98252 | 0.99155 | FALSE | TRUE |
| **Frequency at 150 pa** | CMAP | 0.97048 | 0.99095 | FALSE | 0.49609 | 0.93675 | FALSE | TRUE |
| **Frequency at 200 pa** | CMAP | 0.67398 | 0.95518 | FALSE | 0.69979 | 0.95285 | FALSE | TRUE |
| **Frequency at 250 pa** | CMAP | 0.4085 | 0.92553 | FALSE | 0.72123 | 0.95285 | FALSE | TRUE |
| **Frequency at 300 pa** | CMAP | 0.26029 | 0.88823 | FALSE | 0.48679 | 0.93675 | FALSE | TRUE |
| **Capacitance** | CMAP | 0.93826 | 0.99095 | FALSE | 0.95804 | 0.99155 | FALSE | TRUE |
| **TAU** | CMAP | 0.58258 | 0.95471 | FALSE | 0.57247 | 0.94156 | FALSE | TRUE |
| **EPSC Amplitude** | CMAP | 0.97827 | 0.99095 | FALSE | 0.97166 | 0.99155 | FALSE | TRUE |
| **EPSC Halfwidth** | CMAP | 0.02618 | 0.6545 | FALSE | 0.02173 | 0.64545 | FALSE | TRUE |
| **EPSC TAU** | CMAP | 0.44472 | 0.92553 | FALSE | 0.32205 | 0.91524 | FALSE | TRUE |
| **EPSC PPR** | CMAP | 0.13925 | 0.84087 | FALSE | 0.58182 | 0.94735 | FALSE | TRUE |
| **IPSC Amplitude** | CMAP | 0.79694 | 0.96716 | FALSE | 0.07484 | 0.86716 | FALSE | TRUE |
| **IPSC Halfwidth** | CMAP | 0.75064 | 0.95639 | FALSE | 0.72463 | 0.95285 | FALSE | TRUE |
| **IPSC TAU** | CMAP | 0.64397 | 0.95518 | FALSE | 0.68686 | 0.95285 | FALSE | TRUE |
| **IPSC PPR** | CMAP | 0.23729 | 0.87239 | FALSE | 0.44884 | 0.93675 | FALSE | TRUE |
| **Cranial MEP** | CMAP | 0.30713 | 0.89335 | FALSE | 0.32109 | 0.91524 | FALSE | TRUE |
| **RIN** | SMUP | 0.59076 | 0.95471 | FALSE | 0.67695 | 0.95285 | FALSE | TRUE |
| **RMP** | SMUP | 0.79061 | 0.96416 | FALSE | 0.7043 | 0.95285 | FALSE | TRUE |
| **Sag Ratio** | SMUP | 0.91309 | 0.98583 | FALSE | 0.99051 | 0.99155 | FALSE | TRUE |
| **AP amplitude** | SMUP | 0.42298 | 0.92553 | FALSE | 0.3111 | 0.91524 | FALSE | TRUE |
| **AP threshold** | SMUP | 0.04162 | 0.738 | FALSE | 0.03502 | 0.67346 | FALSE | TRUE |
| **AP halfwidth** | SMUP | 0.45595 | 0.92553 | FALSE | 0.48736 | 0.93675 | FALSE | TRUE |
| **AHP** | SMUP | 0.85581 | 0.97695 | FALSE | 0.87795 | 0.96417 | FALSE | TRUE |
| **AP-RMP** | SMUP | 0.5312 | 0.95471 | FALSE | 0.53727 | 0.93675 | FALSE | TRUE |
| **Frequency at 50 pa** | SMUP | 0.02039 | 0.56639 | FALSE | 0.021 | 0.64545 | FALSE | TRUE |
| **Frequency at 100 pa** | SMUP | 0.16309 | 0.84757 | FALSE | 0.20507 | 0.89521 | FALSE | TRUE |
| **Frequency at 150 pa** | SMUP | 0.03196 | 0.66583 | FALSE | 0.02688 | 0.64545 | FALSE | TRUE |
| **Frequency at 200 pa** | SMUP | 0.10234 | 0.78293 | FALSE | 0.12474 | 0.86716 | FALSE | TRUE |
| **Frequency at 250 pa** | SMUP | 0.26239 | 0.88823 | FALSE | 0.16465 | 0.86716 | FALSE | TRUE |
| **Frequency at 300 pa** | SMUP | 0.39352 | 0.92553 | FALSE | 0.33684 | 0.91524 | FALSE | TRUE |
| **Capacitance** | SMUP | 0.12092 | 0.81703 | FALSE | 0.49067 | 0.93675 | FALSE | TRUE |
| **TAU** | SMUP | 0.74285 | 0.95639 | FALSE | 0.88318 | 0.96417 | FALSE | TRUE |
| **EPSC Amplitude** | SMUP | 0.47411 | 0.92553 | FALSE | 0.36099 | 0.91524 | FALSE | TRUE |
| **EPSC Halfwidth** | SMUP | 0.45117 | 0.92553 | FALSE | 0.67067 | 0.95285 | FALSE | TRUE |
| **EPSC TAU** | SMUP | 0.72352 | 0.95639 | FALSE | 0.94296 | 0.9905 | FALSE | TRUE |
| **EPSC PPR** | SMUP | 0.92668 | 0.98583 | FALSE | 0.9343 | 0.98555 | FALSE | TRUE |
| **IPSC Amplitude** | SMUP | 0.16735 | 0.84757 | FALSE | 0.16798 | 0.86716 | FALSE | TRUE |
| **IPSC Halfwidth** | SMUP | 0.46735 | 0.92553 | FALSE | 0.81927 | 0.96417 | FALSE | TRUE |
| **IPSC TAU** | SMUP | 0.29629 | 0.89335 | FALSE | 0.21669 | 0.90288 | FALSE | TRUE |
| **IPSC PPR** | SMUP | 0.60012 | 0.95471 | FALSE | 0.78134 | 0.95285 | FALSE | TRUE |
| **Cranial MEP** | SMUP | 0.6756 | 0.95518 | FALSE | 0.84163 | 0.96417 | FALSE | TRUE |
| **RIN** | MUNE | 0.1343 | 0.84087 | FALSE | 0.50968 | 0.93675 | FALSE | TRUE |
| **RMP** | MUNE | 0.57913 | 0.95471 | FALSE | 0.58736 | 0.94735 | FALSE | TRUE |
| **Sag Ratio** | MUNE | 0.22536 | 0.84757 | FALSE | 0.50141 | 0.93675 | FALSE | TRUE |
| **AP amplitude** | MUNE | 0.2197 | 0.84757 | FALSE | 0.16361 | 0.86716 | FALSE | TRUE |
| **AP threshold** | MUNE | 0.07847 | 0.75452 | FALSE | 0.11505 | 0.86716 | FALSE | TRUE |
| **AP halfwidth** | MUNE | 0.62422 | 0.95471 | FALSE | 0.52755 | 0.93675 | FALSE | TRUE |
| **AHP** | MUNE | 0.72193 | 0.95639 | FALSE | 0.70502 | 0.95285 | FALSE | TRUE |
| **AP-RMP** | MUNE | 0.61002 | 0.95471 | FALSE | 0.60329 | 0.95227 | FALSE | TRUE |
| **Frequency at 50 pa** | MUNE | 0.89667 | 0.98254 | FALSE | 0.12877 | 0.86716 | FALSE | TRUE |
| **Frequency at 100 pa** | MUNE | 0.75616 | 0.95639 | FALSE | 0.37536 | 0.91524 | FALSE | TRUE |
| **Frequency at 150 pa** | MUNE | 0.18974 | 0.84757 | FALSE | 0.13953 | 0.86716 | FALSE | TRUE |
| **Frequency at 200 pa** | MUNE | 0.68982 | 0.95518 | FALSE | 0.35612 | 0.91524 | FALSE | TRUE |
| **Frequency at 250 pa** | MUNE | 0.57721 | 0.95471 | FALSE | 0.43943 | 0.93675 | FALSE | TRUE |
| **Frequency at 300 pa** | MUNE | 0.31346 | 0.89335 | FALSE | 0.86891 | 0.96417 | FALSE | TRUE |
| **Capacitance** | MUNE | 0.1382 | 0.84087 | FALSE | 0.56673 | 0.94156 | FALSE | TRUE |
| **TAU** | MUNE | 0.88238 | 0.98054 | FALSE | 0.51699 | 0.93675 | FALSE | TRUE |
| **EPSC Amplitude** | MUNE | 0.70808 | 0.95639 | FALSE | 0.72174 | 0.95285 | FALSE | TRUE |
| **EPSC Halfwidth** | MUNE | 0.21848 | 0.84757 | FALSE | 0.21056 | 0.89521 | FALSE | TRUE |
| **EPSC TAU** | MUNE | 0.75034 | 0.95639 | FALSE | 0.87573 | 0.96417 | FALSE | TRUE |
| **EPSC PPR** | MUNE | 0.31732 | 0.89335 | FALSE | 0.16004 | 0.86716 | FALSE | TRUE |
| **IPSC Amplitude** | MUNE | 0.11687 | 0.8116 | FALSE | 0.0284 | 0.64545 | FALSE | TRUE |
| **IPSC Halfwidth** | MUNE | 0.95137 | 0.99095 | FALSE | 0.75894 | 0.95285 | FALSE | TRUE |
| **IPSC TAU** | MUNE | 0.69771 | 0.95627 | FALSE | 0.79205 | 0.96123 | FALSE | TRUE |
| **IPSC PPR** | MUNE | 0.82535 | 0.97695 | FALSE | 0.71729 | 0.95285 | FALSE | TRUE |
| **Cranial MEP** | MUNE | 0.39884 | 0.92553 | FALSE | 0.89679 | 0.96637 | FALSE | TRUE |
| **RIN** | RNS | 0.10614 | 0.78293 | FALSE | 0.155 | 0.86716 | FALSE | TRUE |
| **RMP** | RNS | 0.68978 | 0.95518 | FALSE | 0.32067 | 0.91524 | FALSE | TRUE |
| **Sag Ratio** | RNS | 0.19255 | 0.84757 | FALSE | 0.25609 | 0.91461 | FALSE | TRUE |
| **AP amplitude** | RNS | 0.10343 | 0.78293 | FALSE | 0.10659 | 0.86716 | FALSE | TRUE |
| **AP threshold** | RNS | 0.44694 | 0.92553 | FALSE | 0.99155 | 0.99155 | FALSE | TRUE |
| **AP halfwidth** | RNS | 0.65986 | 0.95518 | FALSE | 0.71235 | 0.95285 | FALSE | TRUE |
| **AHP** | RNS | 0.36882 | 0.92553 | FALSE | 0.84438 | 0.96417 | FALSE | TRUE |
| **AP-RMP** | RNS | 0.70632 | 0.95639 | FALSE | 0.79929 | 0.96417 | FALSE | TRUE |
| **Frequency at 50 pa** | RNS | 0.32061 | 0.89335 | FALSE | 0.31103 | 0.91524 | FALSE | TRUE |
| **Frequency at 100 pa** | RNS | 0.59166 | 0.95471 | FALSE | 0.75414 | 0.95285 | FALSE | TRUE |
| **Frequency at 150 pa** | RNS | 0.77417 | 0.96197 | FALSE | 0.52227 | 0.93675 | FALSE | TRUE |
| **Frequency at 200 pa** | RNS | 0.86168 | 0.97918 | FALSE | 0.74615 | 0.95285 | FALSE | TRUE |
| **Frequency at 250 pa** | RNS | 0.63011 | 0.95471 | FALSE | 0.6091 | 0.95227 | FALSE | TRUE |
| **Frequency at 300 pa** | RNS | 0.59538 | 0.95471 | FALSE | 0.59166 | 0.94817 | FALSE | TRUE |
| **Capacitance** | RNS | 0.90524 | 0.98396 | FALSE | 0.89494 | 0.96637 | FALSE | TRUE |
| **TAU** | RNS | 0.66889 | 0.95518 | FALSE | 0.86649 | 0.96417 | FALSE | TRUE |
| **EPSC Amplitude** | RNS | 0.42407 | 0.92553 | FALSE | 0.37046 | 0.91524 | FALSE | TRUE |
| **EPSC Halfwidth** | RNS | 0.88396 | 0.98054 | FALSE | 0.81603 | 0.96417 | FALSE | TRUE |
| **EPSC TAU** | RNS | 0.17425 | 0.84757 | FALSE | 0.30422 | 0.91524 | FALSE | TRUE |
| **EPSC PPR** | RNS | 0.4665 | 0.92553 | FALSE | 0.49097 | 0.93675 | FALSE | TRUE |
| **IPSC Amplitude** | RNS | 0.52217 | 0.94596 | FALSE | 0.63906 | 0.95285 | FALSE | TRUE |
| **IPSC Halfwidth** | RNS | 0.28515 | 0.89335 | FALSE | 0.16548 | 0.86716 | FALSE | TRUE |
| **IPSC TAU** | RNS | 0.92562 | 0.98583 | FALSE | 0.82755 | 0.96417 | FALSE | TRUE |
| **IPSC PPR** | RNS | 0.04776 | 0.74625 | FALSE | 0.05082 | 0.86716 | FALSE | TRUE |
| **Cranial MEP** | RNS | 0.61743 | 0.95471 | FALSE | 0.45618 | 0.93675 | FALSE | TRUE |
| **RIN** | Cervical MEP | 0.75746 | 0.95639 | FALSE | 0.55081 | 0.93675 | FALSE | TRUE |
| **RMP** | Cervical MEP | 0.05709 | 0.75452 | FALSE | 0.03164 | 0.65917 | FALSE | TRUE |
| **Sag Ratio** | Cervical MEP | 0.10747 | 0.78293 | FALSE | 0.10128 | 0.86716 | FALSE | TRUE |
| **AP amplitude** | Cervical MEP | 0.88715 | 0.98054 | FALSE | 0.47038 | 0.93675 | FALSE | TRUE |
| **AP threshold** | Cervical MEP | 0.01652 | 0.56639 | FALSE | 0.01757 | 0.64545 | FALSE | TRUE |
| **AP halfwidth** | Cervical MEP | 0.30043 | 0.89335 | FALSE | 0.90918 | 0.97135 | FALSE | TRUE |
| **AHP** | Cervical MEP | 0.57486 | 0.95471 | FALSE | 0.58596 | 0.94735 | FALSE | TRUE |
| **AP-RMP** | Cervical MEP | 0.40205 | 0.92553 | FALSE | 0.45204 | 0.93675 | FALSE | TRUE |
| **Frequency at 50 pa** | Cervical MEP | 0.4176 | 0.92553 | FALSE | 0.54982 | 0.93675 | FALSE | TRUE |
| **Frequency at 100 pa** | Cervical MEP | 0.88286 | 0.98054 | FALSE | 0.97332 | 0.99155 | FALSE | TRUE |
| **Frequency at 150 pa** | Cervical MEP | 0.14242 | 0.84087 | FALSE | 0.09866 | 0.86716 | FALSE | TRUE |
| **Frequency at 200 pa** | Cervical MEP | 0.32518 | 0.89335 | FALSE | 0.42067 | 0.93675 | FALSE | TRUE |
| **Frequency at 250 pa** | Cervical MEP | 0.60383 | 0.95471 | FALSE | 0.67843 | 0.95285 | FALSE | TRUE |
| **Frequency at 300 pa** | Cervical MEP | 0.52063 | 0.94596 | FALSE | 0.5557 | 0.93868 | FALSE | TRUE |
| **Capacitance** | Cervical MEP | 0.37145 | 0.92553 | FALSE | 0.84622 | 0.96417 | FALSE | TRUE |
| **TAU** | Cervical MEP | 0.07615 | 0.75452 | FALSE | 0.74121 | 0.95285 | FALSE | TRUE |
| **EPSC Amplitude** | Cervical MEP | 0.48285 | 0.92553 | FALSE | 0.66255 | 0.95285 | FALSE | TRUE |
| **EPSC Halfwidth** | Cervical MEP | 0.05814 | 0.75452 | FALSE | 0.83414 | 0.96417 | FALSE | TRUE |
| **EPSC TAU** | Cervical MEP | 0.00546 | 0.47 | FALSE | 0.66265 | 0.95285 | FALSE | TRUE |
| **EPSC PPR** | Cervical MEP | 0.47814 | 0.92553 | FALSE | 0.66548 | 0.95285 | FALSE | TRUE |
| **IPSC Amplitude** | Cervical MEP | 0.90001 | 0.98254 | FALSE | 0.18037 | 0.86716 | FALSE | TRUE |
| **IPSC Halfwidth** | Cervical MEP | 0.59739 | 0.95471 | FALSE | 0.53771 | 0.93675 | FALSE | TRUE |
| **IPSC TAU** | Cervical MEP | 0.21216 | 0.84757 | FALSE | 0.16771 | 0.86716 | FALSE | TRUE |
| **IPSC PPR** | Cervical MEP | 0.98008 | 0.99095 | FALSE | 0.1155 | 0.86716 | FALSE | TRUE |
| **Cranial MEP** | Cervical MEP | 0.78833 | 0.96416 | FALSE | 0.46792 | 0.93675 | FALSE | TRUE |
| **RIN** | Twitch | 0.7672 | 0.96197 | FALSE | 0.33274 | 0.91524 | FALSE | TRUE |
| **RMP** | Twitch | 0.1942 | 0.84757 | FALSE | 0.22499 | 0.91461 | FALSE | TRUE |
| **Sag Ratio** | Twitch | 0.15347 | 0.84757 | FALSE | 0.24246 | 0.91461 | FALSE | TRUE |
| **AP amplitude** | Twitch | 0.002 | 0.47 | FALSE | 0.00499 | 0.62375 | FALSE | TRUE |
| **AP threshold** | Twitch | 0.62336 | 0.95471 | FALSE | 0.29243 | 0.91524 | FALSE | TRUE |
| **AP halfwidth** | Twitch | 0.46431 | 0.92553 | FALSE | 0.48575 | 0.93675 | FALSE | TRUE |
| **AHP** | Twitch | 0.95712 | 0.99095 | FALSE | 0.09296 | 0.86716 | FALSE | TRUE |
| **AP-RMP** | Twitch | 0.27258 | 0.89335 | FALSE | 0.45025 | 0.93675 | FALSE | TRUE |
| **Frequency at 50 pa** | Twitch | 0.51552 | 0.94596 | FALSE | 0.27631 | 0.91524 | FALSE | TRUE |
| **Frequency at 100 pa** | Twitch | 0.71932 | 0.95639 | FALSE | 0.25377 | 0.91461 | FALSE | TRUE |
| **Frequency at 150 pa** | Twitch | 0.13767 | 0.84087 | FALSE | 0.15 | 0.86716 | FALSE | TRUE |
| **Frequency at 200 pa** | Twitch | 0.20009 | 0.84757 | FALSE | 0.35699 | 0.91524 | FALSE | TRUE |
| **Frequency at 250 pa** | Twitch | 0.07223 | 0.75452 | FALSE | 0.33753 | 0.91524 | FALSE | TRUE |
| **Frequency at 300 pa** | Twitch | 0.14463 | 0.84087 | FALSE | 0.49833 | 0.93675 | FALSE | TRUE |
| **Capacitance** | Twitch | 0.6806 | 0.95518 | FALSE | 0.68146 | 0.95285 | FALSE | TRUE |
| **TAU** | Twitch | 0.17741 | 0.84757 | FALSE | 0.29685 | 0.91524 | FALSE | TRUE |
| **EPSC Amplitude** | Twitch | 0.83371 | 0.97695 | FALSE | 0.7167 | 0.95285 | FALSE | TRUE |
| **EPSC Halfwidth** | Twitch | 0.97456 | 0.99095 | FALSE | 0.95037 | 0.99155 | FALSE | TRUE |
| **EPSC TAU** | Twitch | 0.85179 | 0.97695 | FALSE | 0.7527 | 0.95285 | FALSE | TRUE |
| **EPSC PPR** | Twitch | 0.45286 | 0.92553 | FALSE | 0.29661 | 0.91524 | FALSE | TRUE |
| **IPSC Amplitude** | Twitch | 0.05206 | 0.75452 | FALSE | 0.02234 | 0.64545 | FALSE | TRUE |
| **IPSC Halfwidth** | Twitch | 0.24773 | 0.88823 | FALSE | 0.47194 | 0.93675 | FALSE | TRUE |
| **IPSC TAU** | Twitch | 0.91498 | 0.98583 | FALSE | 0.29925 | 0.91524 | FALSE | TRUE |
| **IPSC PPR** | Twitch | 0.87832 | 0.98054 | FALSE | 0.97012 | 0.99155 | FALSE | TRUE |
| **Cranial MEP** | Twitch | 0.48213 | 0.92553 | FALSE | 0.64558 | 0.95285 | FALSE | TRUE |
| **RIN** | Tetanic | 0.44188 | 0.92553 | FALSE | 0.80633 | 0.96417 | FALSE | TRUE |
| **RMP** | Tetanic | 0.29373 | 0.89335 | FALSE | 0.11482 | 0.86716 | FALSE | TRUE |
| **Sag Ratio** | Tetanic | 0.35355 | 0.92553 | FALSE | 0.23599 | 0.91461 | FALSE | TRUE |
| **AP amplitude** | Tetanic | 0.26339 | 0.88823 | FALSE | 0.19107 | 0.8685 | FALSE | TRUE |
| **AP threshold** | Tetanic | 0.55582 | 0.95471 | FALSE | 0.51377 | 0.93675 | FALSE | TRUE |
| **AP halfwidth** | Tetanic | 0.31021 | 0.89335 | FALSE | 0.1631 | 0.86716 | FALSE | TRUE |
| **AHP** | Tetanic | 0.81174 | 0.97565 | FALSE | 0.78014 | 0.95285 | FALSE | TRUE |
| **AP-RMP** | Tetanic | 0.5442 | 0.95471 | FALSE | 0.52895 | 0.93675 | FALSE | TRUE |
| **Frequency at 50 pa** | Tetanic | 0.35273 | 0.92553 | FALSE | 0.36373 | 0.91524 | FALSE | TRUE |
| **Frequency at 100 pa** | Tetanic | 0.20196 | 0.84757 | FALSE | 0.45153 | 0.93675 | FALSE | TRUE |
| **Frequency at 150 pa** | Tetanic | 0.75397 | 0.95639 | FALSE | 0.81345 | 0.96417 | FALSE | TRUE |
| **Frequency at 200 pa** | Tetanic | 0.97029 | 0.99095 | FALSE | 0.9547 | 0.99155 | FALSE | TRUE |
| **Frequency at 250 pa** | Tetanic | 0.568 | 0.95471 | FALSE | 0.9084 | 0.97135 | FALSE | TRUE |
| **Frequency at 300 pa** | Tetanic | 0.87025 | 0.98054 | FALSE | 0.80524 | 0.96417 | FALSE | TRUE |
| **Capacitance** | Tetanic | 0.3615 | 0.92553 | FALSE | 0.51087 | 0.93675 | FALSE | TRUE |
| **TAU** | Tetanic | 0.78399 | 0.96416 | FALSE | 0.73797 | 0.95285 | FALSE | TRUE |
| **EPSC Amplitude** | Tetanic | 0.18076 | 0.84757 | FALSE | 0.18799 | 0.8685 | FALSE | TRUE |
| **EPSC Halfwidth** | Tetanic | 0.10961 | 0.78293 | FALSE | 0.12107 | 0.86716 | FALSE | TRUE |
| **EPSC TAU** | Tetanic | 0.21343 | 0.84757 | FALSE | 0.29635 | 0.91524 | FALSE | TRUE |
| **EPSC PPR** | Tetanic | 0.40761 | 0.92553 | FALSE | 0.15048 | 0.86716 | FALSE | TRUE |
| **IPSC Amplitude** | Tetanic | 0.77727 | 0.96197 | FALSE | 0.76012 | 0.95285 | FALSE | TRUE |
| **IPSC Halfwidth** | Tetanic | 0.76979 | 0.96197 | FALSE | 0.25307 | 0.91461 | FALSE | TRUE |
| **IPSC TAU** | Tetanic | 0.50405 | 0.93343 | FALSE | 0.53784 | 0.93675 | FALSE | TRUE |
| **IPSC PPR** | Tetanic | 0.69155 | 0.95518 | FALSE | 0.73603 | 0.95285 | FALSE | TRUE |
| **Cranial MEP** | Tetanic | 0.01368 | 0.56639 | FALSE | 0.02653 | 0.64545 | FALSE | TRUE |
| **RIN** | ROTAROD | 0.94692 | 0.99095 | FALSE | 0.82582 | 0.96417 | FALSE | TRUE |
| **RMP** | ROTAROD | 0.06678 | 0.75452 | FALSE | 0.13098 | 0.86716 | FALSE | TRUE |
| **Sag Ratio** | ROTAROD | 0.46354 | 0.92553 | FALSE | 0.34514 | 0.91524 | FALSE | TRUE |
| **AP amplitude** | ROTAROD | 0.60662 | 0.95471 | FALSE | 0.75247 | 0.95285 | FALSE | TRUE |
| **AP threshold** | ROTAROD | 0.75237 | 0.95639 | FALSE | 0.77186 | 0.95285 | FALSE | TRUE |
| **AP halfwidth** | ROTAROD | 0.65968 | 0.95518 | FALSE | 0.54596 | 0.93675 | FALSE | TRUE |
| **AHP** | ROTAROD | 0.85534 | 0.97695 | FALSE | 0.68653 | 0.95285 | FALSE | TRUE |
| **AP-RMP** | ROTAROD | 0.20551 | 0.84757 | FALSE | 0.07602 | 0.86716 | FALSE | TRUE |
| **Frequency at 50 pa** | ROTAROD | 0.73766 | 0.95639 | FALSE | 0.85668 | 0.96417 | FALSE | TRUE |
| **Frequency at 100 pa** | ROTAROD | 0.04428 | 0.738 | FALSE | 0.12475 | 0.86716 | FALSE | TRUE |
| **Frequency at 150 pa** | ROTAROD | 0.22715 | 0.84757 | FALSE | 0.24099 | 0.91461 | FALSE | TRUE |
| **Frequency at 200 pa** | ROTAROD | 0.09792 | 0.78293 | FALSE | 0.36557 | 0.91524 | FALSE | TRUE |
| **Frequency at 250 pa** | ROTAROD | 0.00601 | 0.47 | FALSE | 0.13851 | 0.86716 | FALSE | TRUE |
| **Frequency at 300 pa** | ROTAROD | 0.07209 | 0.75452 | FALSE | 0.33613 | 0.91524 | FALSE | TRUE |
| **Capacitance** | ROTAROD | 0.29914 | 0.89335 | FALSE | 0.23657 | 0.91461 | FALSE | TRUE |
| **TAU** | ROTAROD | 0.31429 | 0.89335 | FALSE | 0.97931 | 0.99155 | FALSE | TRUE |
| **EPSC Amplitude** | ROTAROD | 0.25621 | 0.88823 | FALSE | 0.33524 | 0.91524 | FALSE | TRUE |
| **EPSC Halfwidth** | ROTAROD | 0.68955 | 0.95518 | FALSE | 0.91855 | 0.97304 | FALSE | TRUE |
| **EPSC TAU** | ROTAROD | 0.68474 | 0.95518 | FALSE | 0.64451 | 0.95285 | FALSE | TRUE |
| **EPSC PPR** | ROTAROD | 0.26647 | 0.88823 | FALSE | 0.1717 | 0.86716 | FALSE | TRUE |
| **IPSC Amplitude** | ROTAROD | 0.09015 | 0.78293 | FALSE | 0.10838 | 0.86716 | FALSE | TRUE |
| **IPSC Halfwidth** | ROTAROD | 0.62808 | 0.95471 | FALSE | 0.98825 | 0.99155 | FALSE | TRUE |
| **IPSC TAU** | ROTAROD | 0.17522 | 0.84757 | FALSE | 0.17512 | 0.86716 | FALSE | TRUE |
| **IPSC PPR** | ROTAROD | 0.32167 | 0.89335 | FALSE | 0.42312 | 0.93675 | FALSE | TRUE |
| **Cranial MEP** | ROTAROD | 0.49154 | 0.92985 | FALSE | 0.25427 | 0.91461 | FALSE | TRUE |
| **RIN** | Grip Strength | 0.17132 | 0.84757 | FALSE | 0.51421 | 0.93675 | FALSE | TRUE |
| **RMP** | Grip Strength | 0.4984 | 0.92985 | FALSE | 0.56902 | 0.94156 | FALSE | TRUE |
| **Sag Ratio** | Grip Strength | 0.89033 | 0.98054 | FALSE | 0.10752 | 0.86716 | FALSE | TRUE |
| **AP amplitude** | Grip Strength | 0.9922 | 0.99358 | FALSE | 0.65573 | 0.95285 | FALSE | TRUE |
| **AP threshold** | Grip Strength | 0.68838 | 0.95518 | FALSE | 0.50276 | 0.93675 | FALSE | TRUE |
| **AP halfwidth** | Grip Strength | 0.08761 | 0.78293 | FALSE | 0.01904 | 0.64545 | FALSE | TRUE |
| **AHP** | Grip Strength | 0.96824 | 0.99095 | FALSE | 0.96822 | 0.99155 | FALSE | TRUE |
| **AP-RMP** | Grip Strength | 0.6347 | 0.95518 | FALSE | 0.51502 | 0.93675 | FALSE | TRUE |
| **Frequency at 50 pa** | Grip Strength | 0.99358 | 0.99358 | FALSE | 0.89039 | 0.96637 | FALSE | TRUE |
| **Frequency at 100 pa** | Grip Strength | 0.28353 | 0.89335 | FALSE | 0.77936 | 0.95285 | FALSE | TRUE |
| **Frequency at 150 pa** | Grip Strength | 0.38195 | 0.92553 | FALSE | 0.836 | 0.96417 | FALSE | TRUE |
| **Frequency at 200 pa** | Grip Strength | 0.80585 | 0.97325 | FALSE | 0.26764 | 0.91524 | FALSE | TRUE |
| **Frequency at 250 pa** | Grip Strength | 0.92532 | 0.98583 | FALSE | 0.12306 | 0.86716 | FALSE | TRUE |
| **Frequency at 300 pa** | Grip Strength | 0.49531 | 0.92985 | FALSE | 0.12291 | 0.86716 | FALSE | TRUE |
| **Capacitance** | Grip Strength | 0.54437 | 0.95471 | FALSE | 0.76582 | 0.95285 | FALSE | TRUE |
| **TAU** | Grip Strength | 0.03027 | 0.66583 | FALSE | 0.63913 | 0.95285 | FALSE | TRUE |
| **EPSC Amplitude** | Grip Strength | 0.35416 | 0.92553 | FALSE | 0.37258 | 0.91524 | FALSE | TRUE |
| **EPSC Halfwidth** | Grip Strength | 0.4276 | 0.92553 | FALSE | 0.6103 | 0.95227 | FALSE | TRUE |
| **EPSC TAU** | Grip Strength | 0.98302 | 0.99095 | FALSE | 0.48049 | 0.93675 | FALSE | TRUE |
| **EPSC PPR** | Grip Strength | 0.01937 | 0.56639 | FALSE | 0.00269 | 0.62375 | FALSE | TRUE |
| **IPSC Amplitude** | Grip Strength | 0.69999 | 0.95627 | FALSE | 0.60697 | 0.95227 | FALSE | TRUE |
| **IPSC Halfwidth** | Grip Strength | 0.38967 | 0.92553 | FALSE | 0.53039 | 0.93675 | FALSE | TRUE |
| **IPSC TAU** | Grip Strength | 0.97533 | 0.99095 | FALSE | 0.77344 | 0.95285 | FALSE | TRUE |
| **IPSC PPR** | Grip Strength | 0.61716 | 0.95471 | FALSE | 0.50725 | 0.93675 | FALSE | TRUE |
| **Cranial MEP** | Grip Strength | 0.75392 | 0.95639 | FALSE | 0.44613 | 0.93675 | FALSE | TRUE |
| **RIN** | Weighted pull | 0.09984 | 0.78293 | FALSE | 0.35223 | 0.91524 | FALSE | TRUE |
| **RMP** | Weighted pull | 0.48498 | 0.92553 | FALSE | 0.66373 | 0.95285 | FALSE | TRUE |
| **Sag Ratio** | Weighted pull | 0.21263 | 0.84757 | FALSE | 0.27556 | 0.91524 | FALSE | TRUE |
| **AP amplitude** | Weighted pull | 0.62923 | 0.95471 | FALSE | 0.56561 | 0.94156 | FALSE | TRUE |
| **AP threshold** | Weighted pull | 0.03943 | 0.738 | FALSE | 0.08073 | 0.86716 | FALSE | TRUE |
| **AP halfwidth** | Weighted pull | 0.63946 | 0.95518 | FALSE | 0.64921 | 0.95285 | FALSE | TRUE |
| **AHP** | Weighted pull | 0.22293 | 0.84757 | FALSE | 0.37708 | 0.91524 | FALSE | TRUE |
| **AP-RMP** | Weighted pull | 0.38581 | 0.92553 | FALSE | 0.31358 | 0.91524 | FALSE | TRUE |
| **Frequency at 50 pa** | Weighted pull | 0.94291 | 0.99095 | FALSE | 0.45845 | 0.93675 | FALSE | TRUE |
| **Frequency at 100 pa** | Weighted pull | 0.85149 | 0.97695 | FALSE | 0.21127 | 0.89521 | FALSE | TRUE |
| **Frequency at 150 pa** | Weighted pull | 0.26189 | 0.88823 | FALSE | 0.13519 | 0.86716 | FALSE | TRUE |
| **Frequency at 200 pa** | Weighted pull | 0.74168 | 0.95639 | FALSE | 0.19711 | 0.87996 | FALSE | TRUE |
| **Frequency at 250 pa** | Weighted pull | 0.84984 | 0.97695 | FALSE | 0.24713 | 0.91461 | FALSE | TRUE |
| **Frequency at 300 pa** | Weighted pull | 0.45785 | 0.92553 | FALSE | 0.6565 | 0.95285 | FALSE | TRUE |
| **Capacitance** | Weighted pull | 0.84358 | 0.97695 | FALSE | 0.91341 | 0.97171 | FALSE | TRUE |
| **TAU** | Weighted pull | 0.4359 | 0.92553 | FALSE | 0.61516 | 0.95227 | FALSE | TRUE |
| **EPSC Amplitude** | Weighted pull | 0.33725 | 0.91644 | FALSE | 0.49409 | 0.93675 | FALSE | TRUE |
| **EPSC Halfwidth** | Weighted pull | 0.00752 | 0.47 | FALSE | 0.00776 | 0.64545 | FALSE | TRUE |
| **EPSC TAU** | Weighted pull | 0.19249 | 0.84757 | FALSE | 0.18034 | 0.86716 | FALSE | TRUE |
| **EPSC PPR** | Weighted pull | 0.06804 | 0.75452 | FALSE | 0.06882 | 0.86716 | FALSE | TRUE |
| **IPSC Amplitude** | Weighted pull | 0.21705 | 0.84757 | FALSE | 0.12364 | 0.86716 | FALSE | TRUE |
| **IPSC Halfwidth** | Weighted pull | 0.60225 | 0.95471 | FALSE | 0.68095 | 0.95285 | FALSE | TRUE |
| **IPSC TAU** | Weighted pull | 0.83158 | 0.97695 | FALSE | 0.86504 | 0.96417 | FALSE | TRUE |
| **IPSC PPR** | Weighted pull | 0.84235 | 0.97695 | FALSE | 0.87472 | 0.96417 | FALSE | TRUE |
| **Cranial MEP** | Weighted pull | 0.60967 | 0.95471 | FALSE | 0.18858 | 0.8685 | FALSE | TRUE |
